# Purine nucleoside phosphorylase inhibition is an effective approach for the treatment of chemical hemorrhagic cystitis

**DOI:** 10.1172/jci.insight.176103

**Published:** 2024-01-25

**Authors:** Amanda Wolf-Johnston, Youko Ikeda, Irina Zabbarova, Anthony J. Kanai, Sheldon Bastacky, Robert Moldwin, Joel N.H. Stern, Edwin K. Jackson, Lori A. Birder

**Affiliations:** 1 Renal-Electrolyte Division, Department of Medicine;; 2 Department of Pharmacology and Chemical Biology; and; 3 Department of Pathology, University of Pittsburgh School of Medicine, Pittsburgh, Pennsylvania, USA.; 4 Arthur Smith Institute for Urology, Northwell Health, Zucker School of Medicine at Hofstra/Northwell, Lake Success, New York, USA.

**Keywords:** Inflammation, Urology

## Abstract

Hemorrhagic cystitis may be induced by infection, radiation therapy, or medications or may be idiopathic. Along with hemorrhagic features, symptoms include urinary urgency and frequency, dysuria (painful urination), and visceral pain. Cystitis-induced visceral pain is one of the most challenging types of pain to treat, and an effective treatment would address a major unmet medical need. We assessed the efficacy of a purine nucleoside phosphorylase inhibitor, 8-aminoguanine (8-AG), for the treatment of hemorrhagic/ulcerative cystitis. Lower urinary tract (LUT) function and structure were assessed in adult Sprague-Dawley rats, treated chronically with cyclophosphamide (CYP; sacrificed day 8) and randomized to daily oral treatment with 8-AG (begun 14 days prior to CYP induction) or its vehicle. CYP-treated rats exhibited multiple abnormalities, including increased urinary frequency and neural mechanosensitivity, reduced bladder levels of inosine, urothelial inflammation/damage, and activation of spinal cord microglia, which is associated with pain hypersensitivity. 8-AG treatment of CYP-treated rats normalized all observed histological, structural, biochemical, and physiological abnormalities. In cystitis 8-AG improved function and reduced both pain and inflammation likely by increasing inosine, a tissue-protective purine metabolite. These findings demonstrate that 8-AG has translational potential for reducing pain and preventing bladder damage in cystitis-associated LUT dysfunctions.

## Abstract 2

The purine nucleoside phosphorylase inhibitor 8-aminoguanine reduces pain and prevents damage in a rat model of hemorrhagic cystitis.

## Graphical abstract

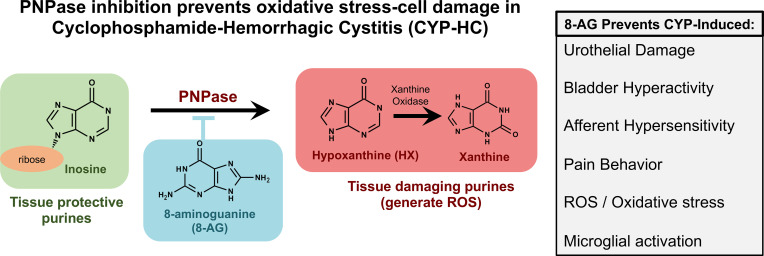

## Introduction

Chronic visceral pain in patients with cystitis (e.g., hemorrhagic/ulcerative cystitis; interstitial cystitis) is among the most difficult types of pain to treat, and the response to treatment is often suboptimal. Hemorrhagic cystitis (HC) is a severe inflammatory condition defined by lower urinary tract signs and symptoms including urinary frequency, hemorrhage, hematuria, and dysuria (painful urination) (1, 2). HC is a complication of infection as well as radiation therapy and chemotherapy. For example, cyclophosphamide (CYP), which is used in the treatment of nonneoplastic disorders and malignant diseases, including pelvic cancers, can induce HC (2–4). In this regard, nitrogen mustard and acrolein (along with up to 150 other metabolite products) are formed in the biotransformation of cyclophosphamide and are highly toxic to epithelial cells lining the bladder lumen, which typically exhibit a slow rate of turnover (5, 6). Acrolein causes increased oxidative stress and release of inflammatory mediators resulting in mucosal edema, hemorrhage, and cell death (7, 8). HC is a cause of substantial morbidity. Despite its high prevalence and impact on quality of life, current management strategies and therapies for HC are few and ineffective once HC has developed; unfortunately, there are no effective strategies for preventing HC.

There is substantive evidence supporting a role for oxidative stress (i.e., uncontrolled increases in the levels of reactive oxygen species, or ROS) in the pathogenesis of multiple inflammatory/injurious conditions. Increased production of ROS occurs in many chronic pain conditions and is involved in mediating pain and inflammation, making ROS and associated oxidative stress important therapeutic targets (9, 10). In this regard, accumulation of oxidative damage over time negatively affects all components of the lower urinary tract (LUT) system (e.g., smooth and striated muscle, nerves, vasculature, collagen and elastin fibers, and epithelium). Emerging evidence, however, suggests that alterations in purine nucleoside phosphorylase (PNPase) activity contribute to oxidative injury and cellular damage (11, 12). PNPase transforms the purine metabolite inosine into hypoxanthine, a purine metabolite associated with the generation of tissue-damaging ROS when hypoxanthine is further metabolized by xanthine oxidase to xanthine and then uric acid (13, 14). Sustained ROS levels are involved in pain pathophysiology and can impair mitochondrial function, leading to oxidative stress–associated cellular damage (15). Here, we report that targeting a single enzyme, PNPase, effectively reduces sensitivity to painful stimuli and reverses bladder dysfunction in a rat model of CYP-induced bladder pain/inflammation.

## Results

**Figure 1:**
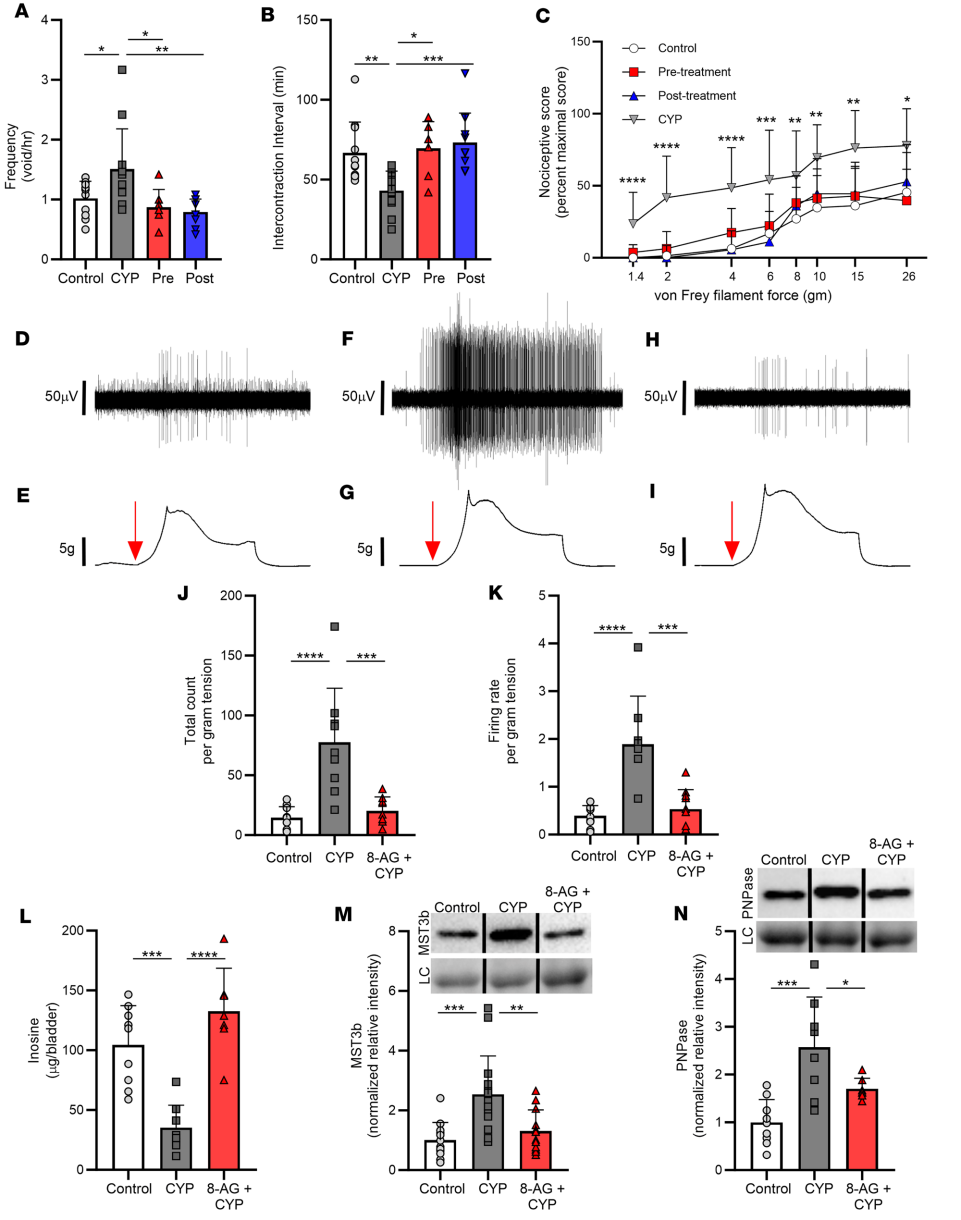
8-Aminoguanine attenuates CYP-related changes in bladder and nerve functions. CYP increased voiding frequency (**A**; *n* = 7–12 per group) and decreased intercontraction interval (**B**; *n* = 7–12 per group). 8-Aminoguanine (8-AG) oral treatment given 14 days prior to the start of CYP (labeled as Pre) or 24 hours after the start of CYP (labeled as Post) prevented (Pre) or restored (Post) these responses to that of control, untreated rats. In addition, CYP increased abdominal (**C**; *n* = 5–23 per group) responses to tactile mechanical stimuli, and this was prevented (Pre) or restored (Post) to that of control, untreated rats by 8-AG treatment. CYP sensitized pelvic nerve afferents innervating the urinary bladder, and this was prevented by oral treatment with 8-AG (**D**–**K**). (**D**, **F**, and **H**) Representative examples of single unit fibers’ responses to bladder distension in bladder preparations from control (**D**; *n* = 3), CYP- (**F**; *n* = 3), or 8-AG + CYP (**H**; *n* = 3) rats. Levels of mechanical stretch were similar in the 3 preparations (**E**, **G**, and **I**). 8-AG decreased the afferent firing total count (**J**; *n* = 3 per group) and frequency (**K**; *n* = 3 per group) of afferents in response to stretch in CYP-bladders. Inosine levels (**L**; *n* = 5–9 per group) were lower in CYP-rat bladders yet were normal in 8-AG + CYP bladders. (**M**) Western immunoblotting of bladder protein lysates from CYP-rat bladders exhibited increases in the inosine-sensitive Ste20-like kinase Mst3b (*n* = 12–15 per group), yet Mst3b expression was similar in control versus 8-AG + CYP-rat bladders. (**N**) 8-AG inhibited the increase in PNPase expression in CYP-rat bladders (*n* = 7–9 per group). (**M** and **N**) Upper insets show representative bands from Mst3b and PNPase Western immunoblotting. Densitometry was normalized to total protein staining (examples shown in lower inset). Representative bands were run on the same blot but were noncontiguous. Data are presented as means ± SD. Ordinary 1-way ANOVA followed by Tukey’s post hoc or Newman-Keuls post hoc multiple-comparison test was used to evaluate significance; *P < 0.05, **P < 0.01; ***P < 0.0001; ****P < 0.00001.

Here, we tested the hypothesis that treatment with CYP would adversely affect voiding and neural function. Bladder cystitis was induced with 3 injections of CYP (75 mg/kg, i.p.) given on days 0, 3, and 6, and testing was performed on day 8. Metabolic cage studies in untreated versus CYP-treated rats (CYP-rats) revealed significant increases in voiding frequency (Figure 1A) and decreases in the intercontraction interval (Figure 1B) in CYP-rats. Also, CYP-rats demonstrated increased von Frey sensitivity (16, 17) to tactile (mechanical) abdominal stimuli (Figure 1C). The von Frey test is a noninvasive behavioral method that uses a series of calibrated von Frey filaments to assess the sensitivity to mechanical stimuli (i.e., response to pressure stimuli) at various anatomical locations (18). These differences indicate a CYP-associated increase in tactile sensitivity, indicative of sensory sensitization. We then addressed the hypothesis that the PNPase inhibitor 8-aminoguanine (8-AG) could prevent or restore CYP-induced changes in both voiding and neural sensitivity. Here, 8-AG was begun 14 days prior to the start of CYP administration (pretreatment group) or 24 hours after start of CYP (posttreatment group). Notably, in CYP-rats treated with oral 8-AG prior to CYP or after the start of CYP with oral 8-AG, both voiding behavior (Figure 1, A and B) and tactile sensitivity (Figure 1C) were similar to that observed in control, untreated rats. Because results were similar in pre- versus posttreatment 8-AG groups, subsequent studies were performed in pretreated animals to better mimic the clinical scenario of pretreatment with 8-AG before administering an injurious chemical, such as CYP, to patients.

We next tested the hypothesis that CYP alters sensitivity of pelvic afferents to bladder stretch, a mechanism that could contribute to CYP-induced increases in mechanosensitivity. Single afferent fibers were recorded from the S1 branch of the bladder-nerve preparation, and responses of afferent fibers were examined after varying degrees of stretch. Bladder sheets were stretched along the base-to-dome axis at a rate of 0.2–0.8 mm per second, held for 30 seconds, and then returned to resting tension. The afferent firing rate was enhanced by mechanical stimulation (Figure 1, D–K). Next, we tested whether CYP-induced changes in neural mechanosensitivity to bladder stretch could be inhibited by 8-AG treatment. CYP sensitized pelvic nerve afferents innervating the urinary bladder, and this was prevented by oral treatment with 8-AG (Figure 1, D–K). Figure 1, D, F, and H, shows representative examples of single fiber responses to bladder distension in bladder-nerve preparations from a control rat (Figure 1D) versus a CYP-rat (Figure 1F) versus a CYP-rat treated with oral 8-AG (Figure 1H). As shown, the firing rate in a bladder afferent from the CYP-inflamed bladder (Figure 1F) was markedly increased as compared with that of an afferent from a control rat bladder (Figure 1D); this response to CYP was blocked in rats treated with 8-AG (Figure 1H). Levels of mechanical stretch were similar in the 3 preparations (Figure 1, E, G, and I). The total count (Figure 1J) or firing frequency (Figure 1K) per gram of tension in CYP-bladders, compared with control bladders, increased more as a function of the level of stretch when held for 30 seconds. Afferent firing ceased as stretch was reversed and bladder tension returned to resting levels. Importantly, 8-AG treatment blocked CYP-induced increases in afferent firing count and rate in response to stretch (Figure 1, J and K).

Inosine, an endogenous purine, is thought to promote antinociception, and PNPase, which is inhibited by 8-AG, metabolizes inosine. Thus, we were motivated to investigate the concept that CYP reduces bladder levels of inosine and that this is prevented by 8-AG. Accordingly, we explored the effects of CYP on bladder levels of inosine in rats pretreated or not with 8-AG. In CYP-bladders, inosine levels were significantly lower compared with control bladders (Figure 1L). Further, in CYP-rat bladders, expression of Mst3b, which is a neuron-specific purine-sensitive (inosine) Ste20-like kinase, was significantly elevated as compared with control urinary bladder (Figure 1M). Mst3b is an important regulator of neuronal axon growth and plays a role in regeneration following inflammation or injury in the mature nervous system (19) Importantly, CYP treatment has been shown to produce increases in urinary bladder neurotrophic factors, including nerve growth factor (NGF), which in turn can activate Mst3b. As shown in Figure 1, L and M, CYP-induced changes in both inosine and Mst3b expression levels were prevented by 8-AG treatment. Moreover, CYP treatment increased expression of PNPase in the bladder, a response that was prevented by 8-AG (Figure 1N).

**Figure 2:**
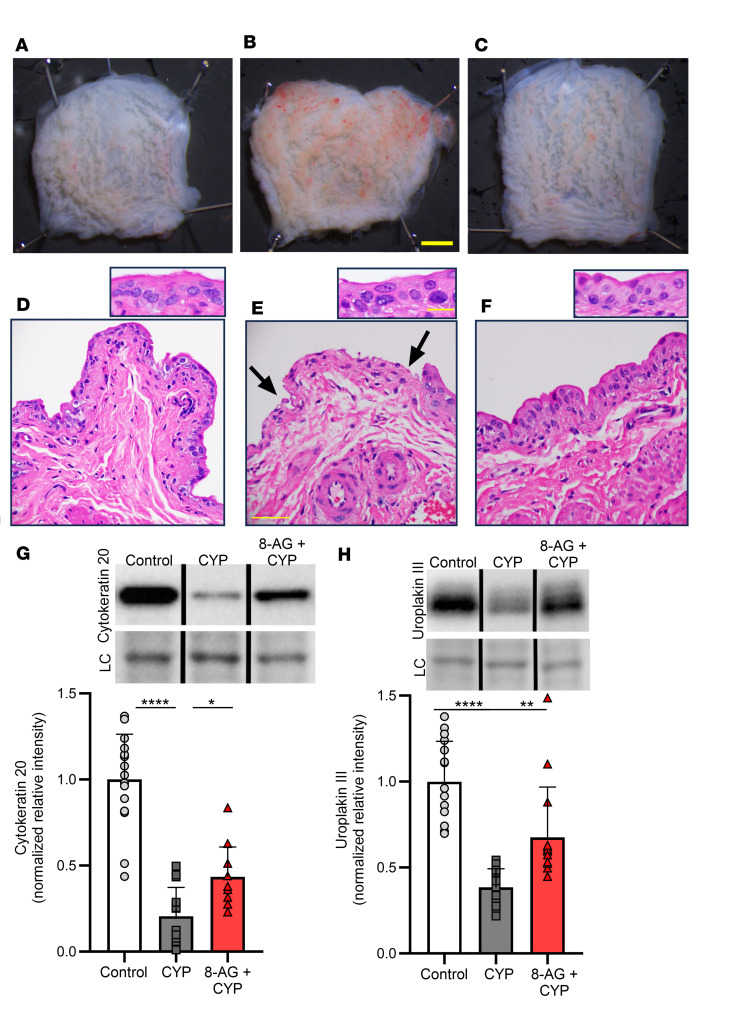
8-AG attenuates CYP-associated morphological and histological changes to the urinary bladder mucosa. (**A**–**F**) Representative images (from 4 experiments) show CYP-associated mucosal hemorrhage (**B**) versus control (**A**) and damage to the apical urothelial surface (shown at arrows) in CYP-bladder (**E**) versus control bladder (**D**). Inset panel (**E**) depicts reactive epithelial cells with large, multinucleated nuclei in CYP-bladders as compared with control. (**G** and **H**) Western immunoblotting revealed decreases in the urothelial umbrella cell proteins cytokeratin 20 (**G**; *n* = 11–16 per group) and uroplakin III (**H**; *n* = 13–16 per group) in CYP-bladders compared with control rat bladders. Upper insets (**G** and **H**) show representative bands from cytokeratin 20 and uroplakin III Western immunoblotting. Densitometry was normalized to total protein staining (loading control [LC]; representative bands shown in lower inset). Representative bands were run on the same blot but were noncontiguous. (**C** and **F**–**H**) 8-AG treatment restored the abnormal morphological and histological changes to a control state. (**A**–**C**) Scale bars: 5 mm. (**D**–**F**) Scale bars: 50 μm and original magnification 40×. (**D**–**F** insets) Scale bars: 20 μm and original magnification 100×. Data are presented as means ± SD. Ordinary 1-way ANOVA followed by Tukey’s post hoc multiple comparison test was used to evaluate significance; *P < 0.05, **P < 0.01; ****P < 0.00001.

As CYP has been associated with bladder inflammation and urothelial damage, we hypothesized that CYP could lead to morphological and histological mucosal alterations. As illustrated in Figure 2, A–F, CYP was associated with morphological and histological damage to the urinary bladder mucosa (20). These changes included mucosal hemorrhage (Figure 2A versus Figure 2B) and necrosis or damage of the surface or apical urothelium (Figure 2D versus Figure 2E) and production of reactive epithelial cells (inset panel Figure 2D versus Figure 2E), which are abnormally large, binucleated and multinucleated cells, as compared with control bladders. These cells are termed reactive as the atypical changes in cellular structure are usually due to an event that occurs close to the cells (inflammation, physical stress). In addition, as shown in Figure 2, G and H, CYP was associated with a significant decrease in markers that are expressed in the urinary bladder superficial epithelium. For example, uroplakins (e.g., uroplakin III or UPIII) are cell membrane proteins that form urothelial plaques covering the apical surface of the urothelium, and cytokeratin 20 is a protein whose expression is restricted to the apical (often termed umbrella) cells (21). Cytokeratin 20 and UPIII expression in the urinary bladder were significantly decreased in CYP-treated rats as compared with control rat bladder (Figure 2, G and H). Given these results, we explored how 8-AG might affect these mucosal alterations. 8-AG treatment prevented all of these morphological and histological abnormalities induced by CYP (Figure 2, C and F–H).

**Figure 3:**
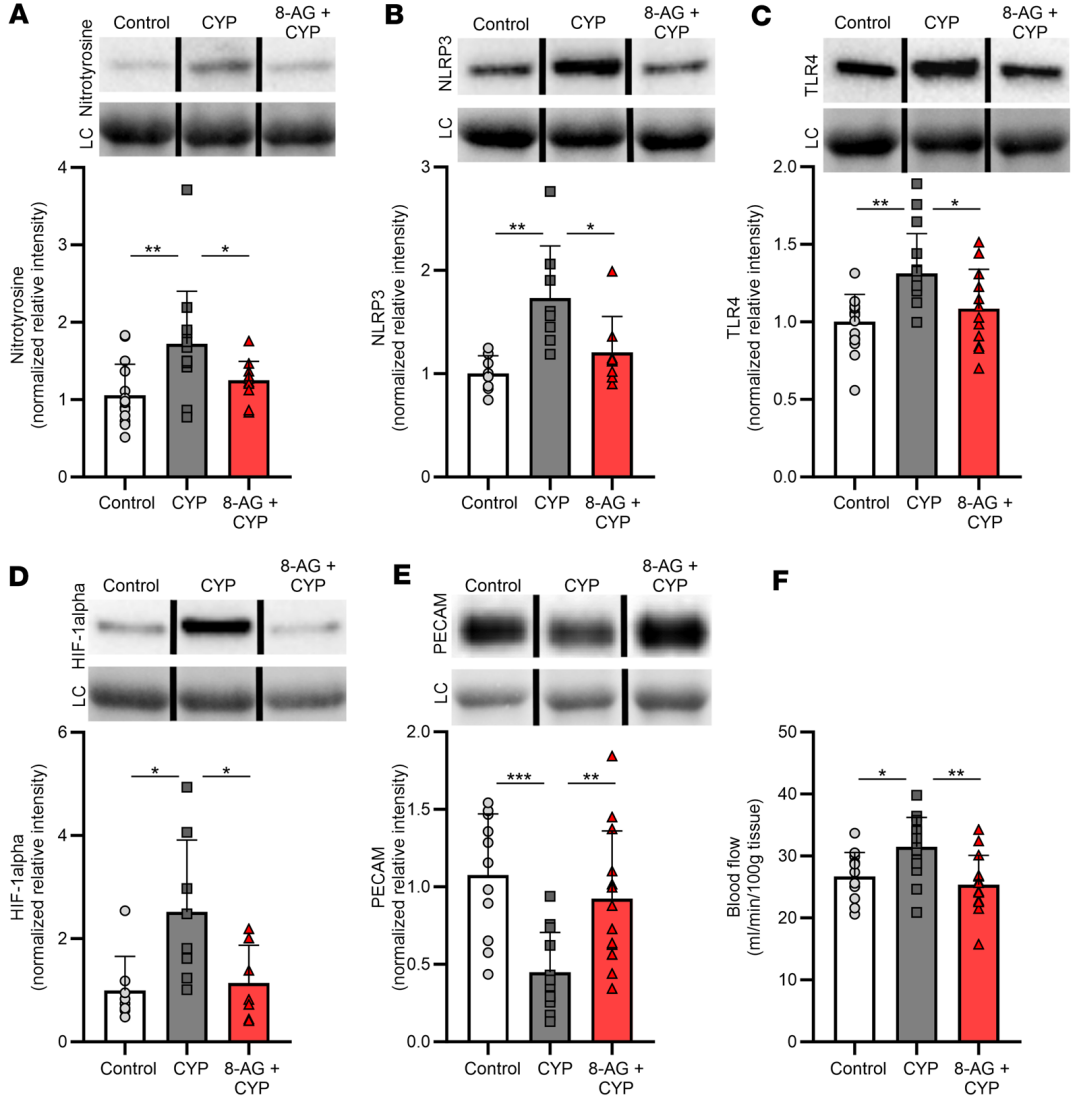
8-AG restores CYP-associated changes in bladder blood flow and proteins linked with oxidative stress and visceral nociception in the rat bladder. (**A**) Western immunoblotting shows significant alterations by CYP in the expression of nitrotyrosine (*n* = 12–15 per group), a biomarker for peroxynitrite action in conditions of cell damage and oxidative stress; (**B**) NLRP3 (*n* = 8 per group), an intracellular sensor that detects danger signals and results in activation of the NLRP3 inflammasome; (**C**) TLR4 (*n* = 12–16 per group), a protein belonging to the pattern recognition receptor family with a key role in amplifying the inflammatory response; (**D**) HIF-1alpha (*n* = 7–8 per group), which plays an important role in inflammatory processes; and (**E**) PECAM-1 (*n* = 11–13 per group), a cellular adhesion and signaling receptor protein expressed at junctions between endothelial cells. (**F**) Doppler flow meter measurements revealing a significant increase in bladder blood flow in CYP-rats compared with control untreated rat bladders (*n* = 12–15 per group). In all cases, treatment with 8-AG blocked CYP-induced changes such that CYP + 8-AG bladders were similar to control, healthy bladders. Upper insets (**A**–**E**) show representative bands from Western immunoblotting. Densitometry was normalized to total protein staining (loading control [LC]; representative bands shown in lower inset). Representative bands were run on the same blot but were noncontiguous. Data are presented as means ± SD. Ordinary 1-way ANOVA followed by either Tukey’s post hoc or Newman-Keuls multiple-comparison test was used to evaluate significance; *P < 0.05, **P < 0.01; ***P < 0.0001.

To further investigate the mechanisms of CYP-induced bladder injury, we examined candidate targets in the urinary bladder that are associated with oxidative stress, inflammation, and visceral nociception. As shown in Figure 3A, compared with control rats, our exploratory findings demonstrated that CYP-rat bladders showed significantly elevated levels of nitrotyrosine, which is an indicator of cell damage, ROS, and inflammation. In this regard, the NOD-like receptor family pyrin domain-containing 3 (NLRP3) inflammasome is a key mediator in triggering inflammatory processes and is thought to contribute to pathogenesis of chronic pain (22, 23). In addition, NLRP3 activation is mediated in part by Toll-like receptors (TLRs) (24), which are pattern recognition receptors that have been reported to play an important role in initiating inflammatory responses (25, 26). Figure 3, B and C, shows an upregulation of both the NLRP3 inflammasome and TLR4 in CYP-rat bladders as compared with control bladders. CYP-rat bladders exhibited increased expression of hypoxia-inducible factor-1alpha (HIF-1alpha, Figure 3D), which plays an essential role in response to hypoxia (which can both induce and be induced by inflammation) in multiple disease states. There was a significant decrease (Figure 3E) in platelet endothelial cell adhesion molecule-1 (PECAM-1, CD31), a cell adhesion receptor expressed at endothelial cell junctions (27). PECAM-1 is involved in maintaining junctional integrity, and alterations in junctional integrity are a sign of inflammation, which can result in increased blood flow to the inflamed/damaged site. As shown in Figure 3F, compared with control rats, CYP-rats showed a significant increase in bladder blood flow (measured using a Doppler flow meter) (28, 29). In rats treated with 8-AG, these CYP-associated abnormalities were completely prevented.

**Figure 4:**
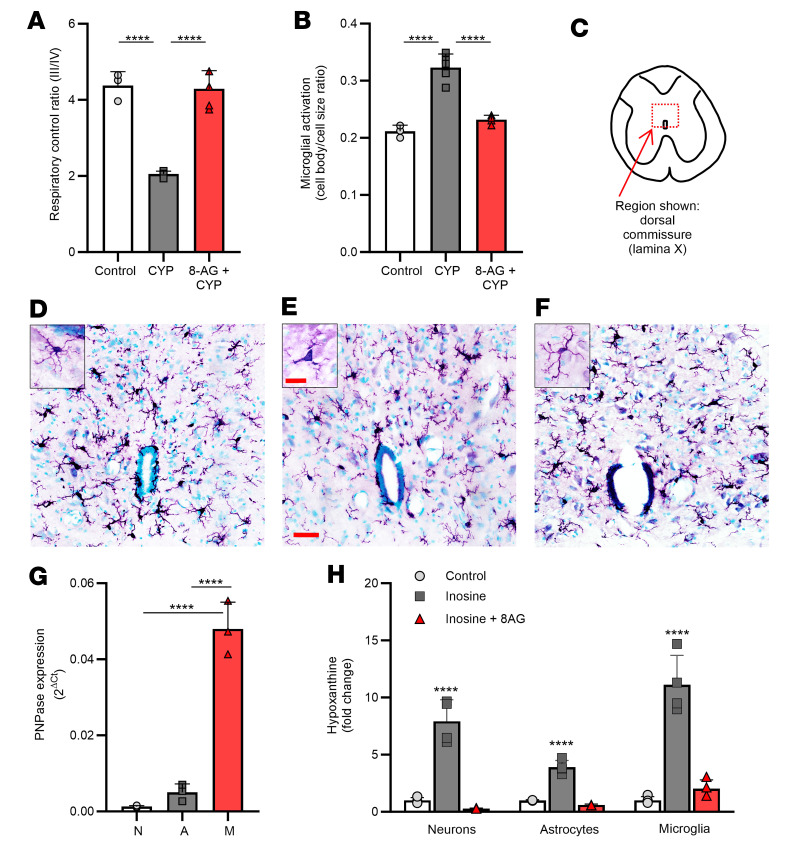
8-AG inhibits CYP-induced changes in mitochondrial respiration and attenuates CYP-induced activation of microglial cells in the L6-S1 spinal cord from CYP-treated rats. (**A**) A decrease in mitochondrial respiratory control ratio (RCR) in mitochondria isolated from CYP-rat L6-S1 spinal cords (SCs) as compared with control SCs (*n* = 3–5 per group). (**B**–**F**) Images were taken of the IBA-1–stained sections (×40 original magnification), and morphological characteristics were analyzed in lamina X (**C**) using Olympus cellSens software. (**B**) CYP significantly increased activation of rat L6-S1 SC microglia as compared with microglia from control rat L6-S1 SCs (*n* = 3–5 per group). (**D**–**F**) Representative images showing CYP-induced morphological changes in microglia from control (**D**), CYP (**E**), or 8-AG + CYP (**F**), whereby activation is exemplified by a retraction of cellular processes and an increase in cell body size. In all cases, 8-AG prevented CYP-induced mitochondrial (**A**) and morphological (**B**–**F**) changes such that mitochondrial RCR and microglia morphology were similar in 8-AG + CYP SCs versus control, healthy SCs. Insets are representative high-magnification images of a single microglial cell; note inset (**E**) depicts microglial activation (e.g., increased cell body size and retraction of processes) following CYP treatment. Scale bars: 20 μm and original magnification 60× (**D**–**F**); inset scale bars: 10 μm and original magnification 100×. (**G**) In cells isolated from neonatal brains, PNPase mRNA expression was highest in microglial cells (bar M, *n* = 3) compared with neurons (bar N, *n* = 3) or astrocytes (bar A, *n* = 3). (**H**) Inosine (substrate for PNPase) was metabolized to hypoxanthine in neurons, astrocytes, and microglia; however, isolated neonatal microglia exhibited the largest increase in hypoxanthine, which was reduced by the PNPase inhibitor 8-AG (*n* = 4 per group). Data are presented as means ± SD. Ordinary 1-way ANOVA followed by Tukey’s post hoc multiple-comparison test was used to evaluate significance; ****P < 0.00001.

Mitochondrial dysfunction has been observed in patients with chronic pain and animal models of chronic pain, and deficits in mitochondrial respiration may contribute to persistent inflammatory pain conditions (30, 31). The mitochondrial respiratory control ratio, or RCR, is an index of how well respiration is coupled to phosphorylation of ADP; RCR decreases with increasing severity of injury or inflammation. Thus, we tested the hypothesis that in CYP-rats mitochondrial RCR is impaired. Figure 4A shows that mitochondria isolated from CYP-rat spinal cords (SCs) exhibited a decreased RCR, meaning an impaired ability of mitochondria to produce energy in the form of ATP, as compared with mitochondria isolated from control rat SCs. By contrast, in 8-AG–treated rats treated with CYP, mitochondrial respiration was similar to that of control rats (Figure 4A).

Glial cells (e.g., microglia) have been linked with both initiation and amplification of persistent pain and central augmentation (32). During inflammation or injury, SC microglial cells become reactive or “activated” as evidenced by retraction of processes often accompanied by an increase in cell body size (32, 33). To measure microglial activation, our study took advantage of a quantitative, semiautomatic image analysis method that has been shown to strongly correlate with qualitative morphological assessment of microglial morphology/activation (34). Using this method, we tested whether CYP treatment in rats was associated with a significant increase in SC microglial activation as compared with control SC microglia. The mechanism of CYP-induced HC is complex and multimodal; however, CYP metabolites (including acrolein) increase ROS (35), which causes damage to bladder cells throughout the bladder wall, in particular the urothelium, which plays an important role in activating underlying bladder nerves. Here, we observed activation of ionized calcium-binding adaptor molecule 1–positive (IBA-1–positive) microglia localized to the L6-S1 SC, a region that receives bladder afferent input from the LUT. In these studies, using quantitative imaging, we assessed the ratio of cell body to cell size as an indicator of microglial activation in the L6-S1 SC dorsal commissure or lamina X (Figure 4C inset panel) near the central canal, a region associated with somatosensory integration and visceral nociception. Figure 4B shows a significant increase in activated IBA-1–positive SC microglia as compared with control SC microglia. Representative images depict morphological changes to SC microglia in the surrounding central canal with CYP treatment (Figure 4E), whereby the processes retract, and cell body size increases, as compared with control SC microglia (Figure 4D; inset panels depict higher magnification of a single SC microglial cell). Moreover, 8-AG prevented CYP-induced changes in SC microglial activation such that microglial morphology in CYP/8-AG rats was similar to that of control rats (Figure 4B and representative image shown in Figure 4F). Reverse transcriptase quantitative polymerase chain reaction (RT-qPCR) revealed that PNPase expression was highest in microglial cells (bar M, Figure 4G) as compared with neurons (bar N, Figure 4G) or astrocytes (bar A, Figure 4G) isolated from neonatal cultures. The ability of PNPase to metabolize the substrate inosine to hypoxanthine was assessed in neonatal cell cultures of neurons, astrocytes, and microglia. As shown in Figure 4H, microglia exhibited the largest increase in hypoxanthine, which was reduced by the PNPase inhibitor 8-AG.

## Discussion

We hypothesize that targeting a non-opioid-based target, namely PNPase, could be an effective therapeutic for the treatment of patients with cystitis. Here, we report results of a treatment that reduces damage to superficial urothelium and urothelial inflammation in rats treated with the cytotoxic agent, CYP. Our findings demonstrate that rats exposed to chronic CYP exhibit 1) bladder voiding dysfunction, 2) increases in tactile sensitivity to mechanical stimuli and single unit afferent nerve firing to bladder stretch, 3) urothelial toxicity, 4) decreased bladder levels of “uro-protective” inosine, and 5) increased SC microglial activation. Importantly, all outcome measures were similar in control rats and CYP-rats treated with 8-AG, an endogenous and potent inhibitor of PNPase. These results demonstrate that the PNPase inhibitor, 8-AG, has strong translational potential for the treatment of patients with cystitis.

PNPase belongs to the family of glycosyltransferases, is expressed in both bacteria and mammals, and is one of the key enzymes involved in the purine salvage pathway (36, 37). PNPase is important for the metabolism of “tissue-protective” purine metabolites (inosine and guanosine) to “tissue-damaging” purines (hypoxanthine and xanthine) that generate free radicals (e.g., ROS) (11, 12, 38). Increased oxidative damage by ROS is deleterious to cells and plays a key role in progression of several diseases. There is substantive evidence that elevated levels of inosine’s downstream metabolite hypoxanthine over time may exhibit harmful effects due to production of ROS when metabolized by xanthine oxidase to xanthine. Increased metabolism of purines has been linked to inflammatory diseases and other disorders, including vascular disease and ischemia (39). For example, a shift in purine catabolism with enhanced accumulation of (potentially injurious) hypoxanthine may play a role in declining myocardial tolerance to ischemia with aging (40). Not surprisingly, treatments that inhibit oxidation of hypoxanthine to xanthine suppress inflammatory cytokines and oxidative stress in a number of disorders. In this regard, xanthine oxidase inhibitors (which suppress metabolism of hypoxanthine to xanthine and xanthine to uric acid) are the standard treatment for gout, characterized by inflammation and joint pain (41). Because PNPase inhibition blocks the metabolism of inosine to hypoxanthine and guanosine to guanine, it is likely the uro-protective effects of PNPase inhibitors in general, and 8-AG in particular, are mediated in part by increases in bladder levels of inosine and guanosine (uro-protective purines) and reductions in bladder levels of hypoxanthine (uro-damaging purine and ROS generator). However, the efficacy of 8-AG on bladder form and function may extend to pleiotropic effects associated with blocking PNPase, including indirectly activating adenosine receptors (which may promote mitochondrial protection and may exert antiinflammatory effects), increasing tissue/cellular protective purines, and reducing damaging purines (thereby reducing sources of ROS) and pathways that impact immune function and inflammation. Moreover, it is conceivable that pleiotropic effects of 8-AG may include mechanisms beyond inhibition of PNPase.

CYP administration alters the properties of bladder afferent nerves located beneath the urothelial lining and within the bladder wall. The location of bladder afferents makes them sensitive to changes that occur in the receptive field (i.e., urine composition; mediators released from the urothelium and other cells embedded within the bladder wall). Afferent nerves are particularly vulnerable to the effects of inflammation and oxidative stress associated with CYP, which, in turn, results in urinary urgency, increased voiding frequency, and pain. CYP-mediated activation of peripheral nerve endings in the urinary bladder has been shown to induce signaling pathways that increase the responsiveness of nerve terminals to mechanical stimulation (42). Our results showing CYP-associated defects in abdominal (visceral) sensory functions, including increased bladder afferent mechanosensitivity, support this view. While the underlying mechanism for these differences may be multimodal, studies in animals have shown that increased free radical damage, oxidative stress, and inflammation may be a contributing factor to CYP cystitis (35, 43).

That CYP treatment in the healthy rat results in increased voiding frequency and a reduction in intervoid interval is consistent with irritative voiding symptoms in patients treated with CYP (44–46). In our studies we examined bladder function in vivo with metabolic cages. A recent study compared a number of functional LUT measurements in rats assessed by metabolic cage experiments versus urodynamic measurements; the results were indistinguishable (47). Advantages offered by this method include the assessment of key variables in conscious rats and the lack of cellular damage caused by catheterization so that downstream ultrastructural, biochemical, and molecular studies could be reliably performed.

Acrolein increases ROS within the urothelium, and free radical–associated cell damage exposes the underlying tissues (vasculature, smooth muscle) to the toxic effects of urine (35). CYP-associated cystitis is likely a result of a complex crosstalk with a number of contributing factors that can include ROS production, oxidative stress, and TLR4 signaling, which in turn can increase the expression of inflammasome components, in particular the NLRP3 inflammasome. Inflammasomes are protein complexes that regulate several cytokines and chemokines and can trigger other cellular processes, causing severe oxidative stress. Importantly, our findings suggest that 8-AG inhibits biomarkers for oxidative nitric oxide damage and inflammation and blocks functional changes associated with irritative bladder inflammation (increased voiding frequency, increased neural sensitivity to mechanical stimuli).

CYP-induced neurotoxicity has been associated with increased peripheral sprouting of bladder sensory afferent nerves, a process driven in part by NGF, a neurotrophin associated with increased nerve sprouting and pain responses in a number of chronic pain conditions, including those involving the LUT (48–51). The protein kinase Mst3b is activated by NGF and is elevated in CYP-rat bladders; yet, Mst3b levels are normal in CYP-rat bladders from rats treated with 8-AG. This may contribute to the ability of 8-AG to reduce pain perception.

There is strong evidence that the neuroprotective purine inosine exhibits antiinflammatory, antinociceptive, and neuroprotective effects to various target organ systems, including the LUT. The mechanism of action for these protective effects may involve stimulation of adenosine receptors and prevention of oxidative damage via scavenging of free radicals and peroxynitrite. Inosine has been shown to attenuate pain responses via both adenosine A_1A_ and A_2A_ receptors in chronic neuropathic and inflammatory pain models (52, 53). Remarkably, inosine has been shown to stimulate neural axonal outgrowth and regeneration of connections after sciatic nerve injury as well as stroke (54, 55). Our findings show that 8-AG prevents detrimental effects of CYP on urinary frequency, single afferent unit hypersensitivity, and pain-related behavior, likely by a pleiotropic mechanism. Though there is clinical evidence that hyperinnervation may be a causal factor in symptoms of bladder cystitis, whether the beneficial effect of 8-AG on voiding and sensory function extends to aberrant sensory sprouting or other mechanisms will need to be clarified in future studies.

The urothelium forms the interface between the urinary space and underlying vasculature and connective, nervous, and muscular tissues. The superficial, or apical surface, layer is composed of large hexagonal cells known as umbrella cells (56). The urothelium plays an important role as a permeability barrier to urine, and this type of intact barrier is a prerequisite for normal afferent signaling from the bladder. The superficial or umbrella cell layer exhibits specialized features that aid the bladder in maintaining normal barrier function, which include specialized proteins called uroplakins that help to prevent proteins as well as ionic and nonionic substances from gaining access to the underlying tissues (6, 56).

The urothelial surface (particularly the uroplakin proteins) is highly vulnerable to exposure to acrolein and other cytotoxic CYP metabolites (57). Over time, the release of inflammatory mediators and increased oxidative stress/ROS can result in negative effects on the underlying vasculature, causing vascular ectasia, damage, and hemorrhage of the lamina propria. In addition, blood flow increases during inflammation, which enables the delivery of oxygen and nutrients to damaged tissues. Consistent with findings in patients who receive CYP therapy (58), our results show that CYP-rat bladders exhibit petechial hemorrhage, increased bladder blood flow, and decreased expression of PECAM-1, a cell adhesion and signaling receptor that regulates endothelial junctional integrity. Further, we show evidence of CYP-induced cell exfoliation, mucosal denudation, and decreases in the umbrella cell marker uroplakin and the differentiation marker cytokeratin 20. Also, CYP produces atypical cytological changes, including appearance of binucleated and reactive (i.e., irregular shaped, larger) nuclei. These abnormalities, as well as the inflammation-associated increases in bladder blood flow, were preserved in CYP-rat bladders from rats treated with the PNPase inhibitor 8-AG.

The most abundant cells in the nervous system are represented by glial cells, which can respond to numerous insults and in turn facilitate the development of chronic pain conditions. SC microglia play an important role in driving the creation and maintenance of allodynia and hyperalgesia and are therefore critical to a number of chronic pain syndromes. (59–62). For example, persistent activation of SC microglial cells due to damaged or persistent inflammation of the viscera may contribute to both initiation and amplification of persistent pain and central augmentation (60, 62–64). Microglial cells have been shown to undergo structural and functional modifications in the SC in animal models of chronic pain. Here, we show that control (i.e., resting) SC microglial cells extend ramified processes. This cellular morphology enables microglia to constantly surveil their environment. However, after administration of CYP, SC microglia (which highly express the enzyme PNPase) become activated and change from a ramified to an amoeboid shape with enlarged cell bodies and shortened processes. The mechanisms that are involved in activation of SC glial cells in CYP-rat bladders are likely to involve complex signaling pathways that will require a comparative set of experiments to determine. However, our findings show changes in bladder afferent excitability with visceral inflammation coupled with changes in glial morphology in SC regions to which bladder afferents project. As SC microglia can respond to extracellular signals, these findings suggest that bladder inflammation promotes activation of bladder afferents and corresponding release of neural mediators that promote microglial activation via a bidirectional neural-glial signaling mechanism, resulting in subsequent pain hypersensitivity. Our findings further show that a PNPase inhibitor attenuates microglial cell activation and associated visceral inflammation and hypersensitivity induced by a bladder-centric model of HC. Taken together, a dysregulation of PNPase (elevated in CYP-bladders) may be an important contributing factor in the initiation (and perhaps the long-lasting maintenance) of pathways resulting in HC-associated bladder pain.

Current management strategies and therapies for HC arising from antineoplastic chemotherapy, such as CYP, include supportive measures (hyperhydration, forced diuresis, and bladder irrigation) (65, 66), as well as intravesical astringents (e.g., the tissue fixative formalin). The medication sodium 2-mercaptoethane-1 sulfonate (Mesna) has been used as prophylaxis for CYP-induced HC. Unfortunately, this agent is ineffective as a treatment once HC has developed and is associated with a number of adverse effects. Furthermore, findings from studies including a retrospective investigation of 718 patients receiving CYP with or without Mesna *do not support its use* as a preventative measure and suggest that Mesna may actually be harmful in such patients (4, 67–69). Clearly, treatment of CYP-associated HC is an unmet medical need with limited to no available options. Perhaps, most importantly, no current or historic therapies are known to prevent the advent of the above-noted histologic or physiologic abnormalities, so the promise of an effective treatment that would avert the adverse events occurring in patients with cystitis is extremely enticing.

HC is a common urological condition associated with high morbidity for which management strategies have been suboptimal. We observed that CYP-induced HC rats, compared with control/untreated rats, exhibit 1) bladder filling/storage and emptying dysfunction, 2) increases in tactile (neural) sensitivity to mechanical stimuli, 3) gross inflammatory changes including urothelial regions of petechial hemorrhages, 4) increased biomarkers associated with pain and oxidative stress, and 5) augmented activation of SC microglia known to contribute to chronic pain states. Importantly, oral dosing of the PNPase inhibitor 8-AG completely prevented these CYP-induced dysfunctions to a control, healthy state. In addition, 8-AG treatment begun after CYP induction of bladder inflammation significantly reduced bladder hyperactivity and pain behavior (mechanical allodynia). This is indeed an important observation as these findings suggest that 8-AG may be effective even in cases once HC has already developed. Regarding clinical relevance, preliminary studies from our research group indicate that patients with cystitis exhibited elevated levels of uro-toxic indicators of oxidative stress as compared with healthy controls (unpublished observations). This suggests that alterations in PNPase may result in a dysregulation of purine metabolism with increased free radical formation and oxidative stress. In addition to the effects on the urinary bladder, 8-AG exhibits wide-ranging beneficial effects on the form and function of other organ systems. In this regard, 8-AG reverses retinal degeneration (70) and increases the life span of hypertensive Dahl SS rats on a high-salt diet by completely preventing strokes (71). Preliminary preclinical experiments have demonstrated initial safety and lack of toxicity with no adverse effects on major organ systems. Though additional studies are required to validate the potential of 8-AG treatment for LUT disorders, these and other findings support the conclusion that 8-substituted amino purines (such as 8-AG) should be included in the drug development pipeline for cystitis-associated bladder dysfunctions.

## Methods

### Sex as a biological variable.

This study employed female young (3 months) Harlan Sprague-Dawley rats from Envigo. The CYP-induced cystitis model is well established using female rats to examine inflammatory and nociceptive pathways. However, we do not rule out the possibility that our findings could be relevant to more than one sex, as CYP-induced HC has been reported to occur in both male and female patients.

### Animals.

Bladder cystitis was induced with 3 injections of 75 mg/kg (i.p.) of CYP (Cayman Chemical) given on days 0, 3, and 6. 8-AG (5 mg/kg/d, Toronto Research Chemicals) was administered in drinking water with daily dosing monitored. This dose of 8-AG was selected based on our preliminary studies showing that 5 mg/kg/d provides a urinary concentration of 8-AG approximately 15 ± 3 (mean ± SEM; *n* = 4) µmol/L, a concentration that is at least 5 times the inhibition constant, or *K_I_*, of 8-AG against PNPase (*K_I_* estimated by us to be 2.8 µmol/L against human recombinant PNPase with inosine as substrate) (72). Animals were randomly assigned into the following groups: 1) vehicle control, 2) CYP treatment only (sacrificed on day 8), and 3) treatment with both 8-AG and CYP, whereby 8-AG is started 14 days prior to the start of CYP. In a separate set of animals, 8-AG treatment was begun 24 hours after the start of CYP. All animals were sacrificed on day 8.

### Voiding analysis.

Control, CYP-treated, and 8-AG–treated rats were placed in metabolic cages on days 7–8 of the CYP dosing regimen. The light cycle was from 7 am to 7 pm, and food and water were provided ad libitum. Voided urine was captured and measured on a plate/load cell system controlled by a comprehensive lab animal monitoring system (Columbus Instruments). Data were analyzed on LabChart software (ADInstruments), averaged for 24 hours, and analyzed for 12-hour periods during the day (7 am—7 pm) and night (7 pm—7 am). Voiding frequency (voids per hour), intervoid interval, and volume per void were analyzed. Voiding frequency was calculated as the number of voiding events per hour during 24 hours and during the 12-hour day and 12-hour night periods. Volume per void, which defines bladder capacity, was calculated as an average of the voids occurring during these periods.

### Von Frey testing.

On day 7–8 of the CYP protocol, pain behaviors were assessed by scoring animals’ behaviors in response to application of von Frey filaments following a testing protocol developed by Auge et al. (18). Baseline testing was performed prior to any treatments (CYP or 8-AG). Testing was performed by a single experimenter. Rats were placed in individual transparent cages placed on top of an elevated mesh stand support (Bioseb) and were allowed to acclimate for a minimum of 30 minutes, until each rat was resting quietly. Increasing filaments within a set of 8 (1.4, 2, 4, 6, 8, 10, 15, and 26 g, Stoelting Co.) were each applied 3 times, with a minimum of 5 seconds between each test. Each instance was scored on a scale as follows: 0 = no response, 1 = reaction of the animal, 2 = reaction and change of position, or 3 = reaction, change of position, and licking and/or vocalization. Nociceptive score for each filament was calculated as a percentage of the maximal possible score.

### Single unit afferent nerve recordings.

Single unit afferent nerve activity was recorded from the sacral S1 dorsal root fibers of female Sprague-Dawley rats receiving no intervention or treatment with CYP or CYP with 8-AG pretreatment using a previously described methodology (73, 74). Briefly, the urinary bladder was dissected with associated S1 dorsal roots, and the bladder was cut along the ventral aspect to form a sheet. The bladder/nerve preparation was placed into a temperature-controlled organ bath perfused with oxygenated Krebs solution. The base of the bladder was secured with pins to the chamber, and the dome was connected to a tension transducer in line with a computer-controlled micromanipulator. The nerve roots were passed into bilateral oil chambers and split into 10 to 20 fine filaments. The filaments were wrapped around a platinum-iridium recording electrode to measure biphasic depolarization in response to mechanical distention of the bladder. Controlled stretches were applied to the bladder using a computer prompt, varying the distance, speed, and duration of the stretch protocol. The velocity of stretch ranged between 0.2 to 0.8 millimeters/second with a 30-second hold followed by a return to baseline. Recorded units were classified based on amplitude. Mechanosensitivity of individual units was determined by linear regression of total counts or counts/second plotted against mean tension during the 30-second hold.

### Vascular alterations.

On day 8 of the CYP protocol, animals were anesthetized by isoflurane, and the bladder was surgically exposed for assessment of vascular blood perfusion. Real-time blood perfusion (1 mm^3^ tissue) was accomplished using a BLF22D laser Doppler flowmeter (Transonic Systems, Inc) with a surface probe (TypeS-APLPHS) applied to the serosal surface of the bladder (apex and neck) wall using Doppler light shift from moving RBCs to analyze flow by the Bonner algorithm. This method gives robust, noninvasive microvascular flow signals in the bladder wall of anesthetized rats (29).

### Histopathology.

Following blood flow measurements, rats were sacrificed under isoflurane anesthesia. Gross macroscopic observations of bladder petechiae were noted and images taken with an Olympus SZX16 dissecting microscope using cellSens software (Olympus). Bladders were dissected and prepared for histopathology and molecular studies. For histological assessment of urinary bladder, sections of bladders were fixed flat in 10% formalin. After embedding in paraffin, sections were stained with hematoxylin and eosin by the UPMC Research Pathology Core shared resource. Images were collected on an Olympus BX-63 microscope using Olympus cellSens software and assessed by an independent pathologist for morphological changes in the urothelial mucosa.

### Western immunoblotting.

Bladder preparations (including both full wall thickness and mucosa separated from the underlying lamina propria and detrusor smooth muscle) were homogenized using Lysing Matrix D in a FastPrep 24 instrument (MP Biomedicals) in HBSS (5 mM KCl, 0.3 mM KH_2_PO_4_, 138 mM NaCl, 4 mM NaHCO_3_, 0.3 mM Na_2_HPO_4_, 5.6 mM glucose, and 10 mM HEPES, pH 7.4) containing complete protease inhibitor cocktail (1 tablet/10 mL, Roche) and phosphatase inhibitor cocktail (MilliporeSigma, 1:100). After centrifugation (16,200*g*; 15 minutes at 4°C), the membrane protein fraction was prepared by suspending the membrane pellets in lysis buffer containing 0.3 M NaCl, 50 mM Tris-HCl (pH 7.6), 0.5% Triton X-100, and the same concentration of protease inhibitors as above. The suspensions were incubated on ice and centrifuged (16,200*g*; 15 minutes at 4°C). The protein concentrations of the combined supernatants were determined using the Pierce BCA protein assay (Thermo Fisher Scientific). After denaturation (100°C for 5 minutes) in the presence of Laemmli sample buffer, lysate from each sample was separated on a 4%–15% TGX Stain-Free SDS-PAGE gel (Bio-Rad). As a reliable loading control, total protein measurement per sample was determined using Bio-Rad Stain-Free SDS-PAGE technology. Ultraviolet-activated protein fluorescence within the gel was imaged on a ChemiDoc MP (Bio-Rad). After proteins were transferred to PVDF membranes, total protein fluorescence of the membrane-bound proteins was imaged on a ChemiDoc MP (Bio-Rad), and the membranes were incubated in 5% (weight/volume; w/v) dried milk dissolved in TBS-T (20 mM Trizma, 137 mM NaCl, 0.1% Tween 20, pH 7.6), rinsed with TBS-T, and incubated overnight at 4°C with primary antibody: cytokeratin 20 (Abcam AB230524), HIF-1alpha (Abcam AB216842), Mst3b (Cell Signaling Technology 4062S), nitrotyrosine (Enzo Life Science BML-SA468-0100), NLRP3 (Abcam AB214185), PECAM (Novus Biologicals NB100-2284), PNPase (Atlas Antibodies HPA001625), TLR4 (Santa Cruz Biotechnology SC-293072), and UPIII (Abcam AB231576) diluted in TBS-T containing 5% (w/v) milk or 5% (w/v BSA). After washing in TBS-T, the membranes were incubated with secondary antibody (sheep anti-mouse HRP Cytiva, NA931-1ML; or donkey anti-rabbit HRP, Advansta, R-05788-500) for 1 hour in 5% (w/v) milk TBS-T, washed, incubated in WesternBright Quantum (Advansta), and imaged on a ChemiDoc MP (Bio-Rad). Data were quantified and analyzed in Image Lab software (Bio-Rad). The volume (intensity) of each protein species was determined and normalized to total protein imaging of the membrane using Bio-Rad Stain-Free SDS-PAGE technology.

### Measurement of inosine in bladder and hypoxanthine in culture medium.

Inosine in bladder and hypoxanthine in culture medium was measured by ultraperformance liquid chromatography-tandem mass spectrometry as recently described by us (75).

### Microglial histology and activation.

Under isoflurane anesthesia, rats were transcardially perfused with PBS containing 100 U of heparin, followed by 4% paraformaldehyde. SC segments (L6-S1) were sucrose-protected, frozen in optimal cutting temperature compound, and sectioned at 20 μm on a Leica cryostat onto slides. Sections were rinsed in PBS (2.68 mM KCl, 1.47mM KH_2_PO_4_, 137 mM NaCl, 8 mM Na_2_HPO_4_, pH 7.4) and exogenous peroxidase–blocked with 1% hydrogen peroxide and nonspecific staining blocked with 0.5% BSA, then incubated overnight at 4°C with rabbit anti–IBA-1 (Wako 019-19741). After rinsing with PBS, sections were incubated with biotinylated goat anti-rabbit secondary antibody and then avidin-biotin peroxidase (Vectastain ABC kit, Vector Laboratories), visualized with VIP peroxidase substrate (Vector Laboratories), and counterstained with methyl green. Sections were dehydrated through xylene and ethanol gradients before coverslipping with VectaMount permanent mounting medium (Vector Laboratories). Images were taken of the IBA-1–stained sections (×40 original magnification) and morphological characteristics of the stained microglia were analyzed using Olympus cellSens software. In the standard assessment of microglia, the degree of activation is graded based on a visual analysis of morphological properties as described by Kreutzberg (76). Microglia were both qualitatively assessed (e.g., activation exhibits both retraction of processes and increased cell body size) and quantitatively assessed using a modification of previously published methods (69). A minimum of 3 immunohistochemically labeled sections per SC region were analyzed by a researcher blinded for the treatment. Using ImageJ (National Institutes of Health), a standardized region of interest was selected, and the IBA-1–stained section was isolated into a single-color image followed by the threshold function to create a binary image. The total cell size, including the entire cell body plus projections, was measured by the analyze particle function with no size filter. Next, the microglial central cell body was selected, and the analyze particle function was used to measure the cell body size. The cell body–to–cell size ratio was then calculated and used as a quantitative measurement of microglial activation.

### Mitochondrial respiration.

Mitochondria were isolated from control, CYP-treated, and CYP-treated with 8-AG Sprague-Dawley rat SCs as described previously (77) with modifications. Briefly, after deep anesthesia with isoflurane (5%), the SC segments L6-S1 (≈80 mg) were isolated by extrusion (78) and placed in a mitochondrial solution containing 5 mM HEPES, 125 mM KCl, 2 mM Pi, 20 μM EDTA, 5 mM MgCl_2_, and 0.2 mg/mL BSA, adjusted to pH 7.4 with 100 mM KOH. The tissue was then minced by a McIlwain motorized tissue chopper (Brinkmann) set to chop at a 10 μm interval. The minced tissue was placed in 10 mL of mitochondrial solution and homogenized by a few passes with a motorized Teflon pestle. The homogenate was spun at 1,000*g* for 10 minutes. The supernatant was then spun at 10,000*g* for 10 minutes to obtain a second pellet containing the mitochondria. This pellet was resuspended in 100 μL of mitochondrial solution, and 25–50 μL of the suspension was placed in a gas-tight vessel containing a Clark-type oxygen microelectrode (MI-730/OM-4; Microelectrodes) to measure the state 3 (succinate + ADP) and state 4 (succinate alone) respiratory rates. The electrode was calibrated (79) considering a total amount of dissolved O_2_ in aqueous solution after equilibration with air at 36°C to be 215 μM, zeroed with sodium dithionite. The RCR, a measure of the “tightness of coupling” between electron transport and oxidative phosphorylation, was determined from the ratio of state 3 to state 4 rates of respiration. An RCR of 2–4 is considered acceptable for complex II substrates (80).

### Neuronal cultures.

Cortical neurons were harvested from E16–E18 Sprague-Dawley rat embryos as previously described (81, 82). Briefly, both male and female embryonic brains were isolated, trypsinized, triturated, and seeded on 10 cm poly-d-lysine culture plates (BD Biosciences). Neurons were grown in Neurobasal medium (Life Technologies) containing B27 supplement, 0.5 mM glutamine, 100 U/mL penicillin, and 100 μg/mL streptomycin. Neurons (95% pure) were maintained by one-half medium exchange 3 and 5 days after culture. Experiments were conducted on in vitro day 6 (DIV6).

### Astrocyte cultures.

Astrocytes were harvested using our previously published protocol (81, 83). Briefly, brains were isolated from male and female postnatal day 1–2 Sprague-Dawley rat pups. The tissue was isolated, trypsinized, triturated, and seeded onto 75 cm^2^ tissue culture flasks. Cells were grown to 90%–95% confluence in DMEM/F12/10% FBS, 100 U/mL penicillin, and 100 μg/mL streptomycin. Experiments were conducted in 10 cm poly-d-lysine culture plates after several propagations to select astrocytes only.

### Microglia cultures.

Primary microglial cultures were isolated from postnatal day 1–2 male and female Sprague-Dawley rat pups using a modification of the method described by Ni and Aschner (81, 84). In brief, microglia cultures were harvested following the same protocol used to harvest astrocytes, except that the resulting brain cell mix was plated onto 225 cm^2^ flasks (about 4 to 6 brains per flask). Cells were plated and grown in DMEM/F12/10% FBS, 100 U/mL penicillin, and 100 μg/mL streptomycin. The flasks were left untouched for the first week following culture, then given complete medium exchange every 2–3 days for 2 weeks. Three weeks after culture, cells were given a complete medium exchange, and flasks were shaken at low speed for 8 minutes, resulting in the dissociation of microglia cells from the flask into the media. The media were collected and centrifuged for 5 minutes at 200*g* and 4°C. Microglia were plated onto 35 mm poly-d-lysine culture plates at a density of 500,000 cells per plate. Experiments were performed 1 day after plating to avoid growth of astrocytes.

### Validation of cell type enrichment in primary CNS cultures.

DIV6 neurons, DIV1 microglia, and confluent monolayer astrocytes were harvested in RIPA buffer supplemented with EDTA and protease/phosphatase inhibitors. Cell types were characterized by Western immunoblotting using markers for neurons, astrocytes, and microglia as described previously (85).

### RT-qPCR.

Total RNA was isolated from neurons, astrocytes, and microglia using TRIzol reagent (Thermo Fisher Scientific) according to the manufacturer’s instructions. The cDNA was synthesized using iScript cDNA synthesis kit (Bio-Rad). qPCR analysis was performed using Power SYBR Green PCR Master Mix (Thermo Fisher Scientific) in the Applied Biosystems QuantStudio 3 Real-Time PCR System (Thermo Fisher Scientific). Primers for PNPase were 5′-TGATCTGTGGTTCCGGCTTA-3′ (forward) and 5′-CACTGGGAACGTCACCTTG-3′ (reverse); for β-actin 5′-ACTCTTCCAGCCTTCCTTC-3′ (forward) and 5′-ATCTCCTTCTGCATCCTGTC-3′ (reverse). Threshold cycle (Ct) for β-actin was subtracted from Ct for PNPase to calculate 2*^ΔCt^*.

### Statistics.

Data were analyzed in GraphPad Prism 10 by ordinary 1-way ANOVA followed by either Tukey’s post hoc or Newman-Keuls post hoc multiple-comparison test. *P* < 0.05 was considered significant. Results are expressed as means ± SD.

### Study approval.

The Institutional Animal Care and Use Committee of the University of Pittsburgh approved all procedures. The investigation conforms to the *Guide for the Care and Use of Laboratory Animals* published by the US National Institutes of Health (NIH Publication No. 85-23, revised 1996).

### Data availability.

Data are available in the paper’s supplemental material (Supporting Data Values) or from the corresponding author upon request.

## Author contributions

LAB and EKJ designed research studies. AWJ, YI, and IZ conducted experiments. AWJ, YI, and IZ acquired data. LAB, AWJ, YI, IZ, AJK, EKJ, and SB provided reagents and developed methodologies. LAB, AJK, EKJ, AWJ, RM, and JNHS wrote the manuscript. LAB, AJK, EKJ, RM, JNHS, and SB interpreted data.

## Supplementary Material

Unedited blot and gel images

Supporting data values

## Version 1 01/25/2024

In-Press Preview

## Version 2 03/08/2024

Electronic publication
