# Supplementary material for: Purine nucleoside phosphorylase inhibition is an effective approach for the treatment of chemical hemorrhagic cystitis
Source: JCI Insight. 2024 Jan 25;9(5):e176103. doi: 10.1172/jci.insight.176103 (PMC10972598; doi:10.1172/jci.insight.176103)
Supplement: Unedited blot and gel images [file jciinsight-9-176103-s162.pdf]

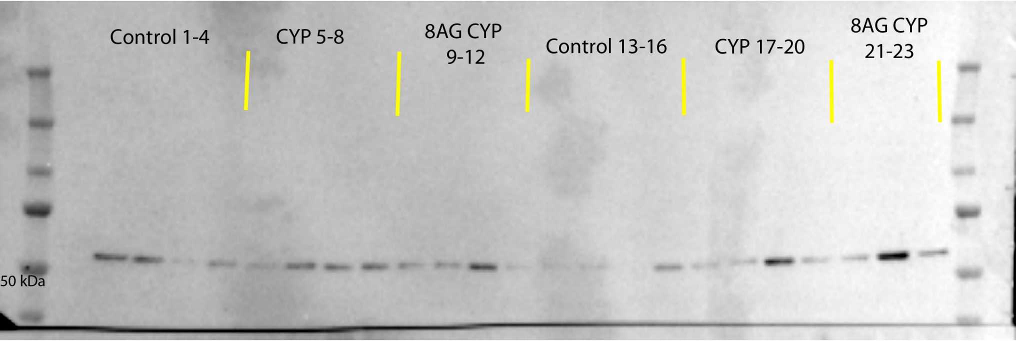

Full unedited blot (1 of 2) for Figure 1M,
MST3b (59kDa), top portion of membrane probed. Cell Signaling 4062, representative bands taken from different blot.

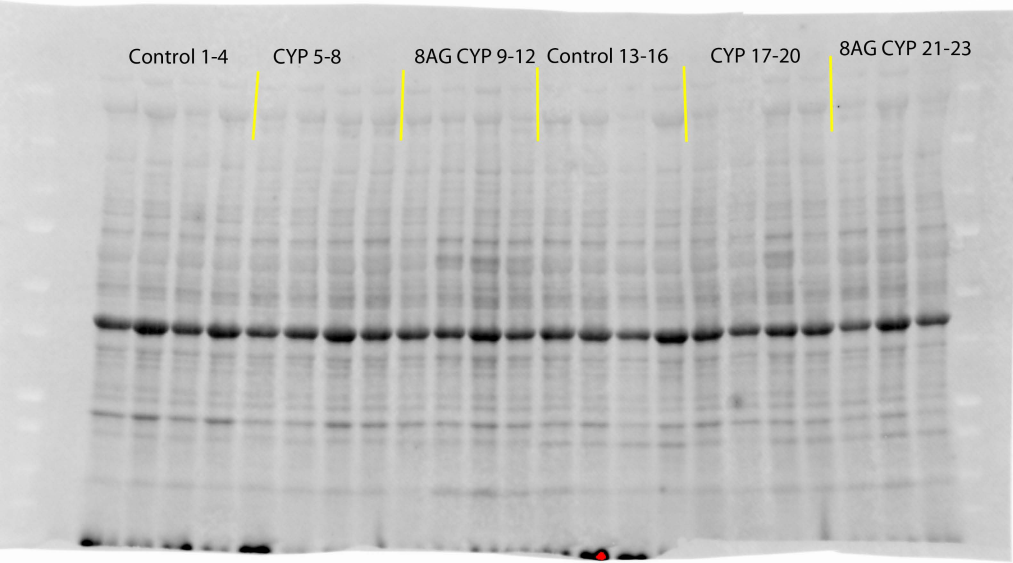

Full unedited stain free blot (1 of 2) for Figure 1M (MST3b)
Representative lanes taken from blot 2

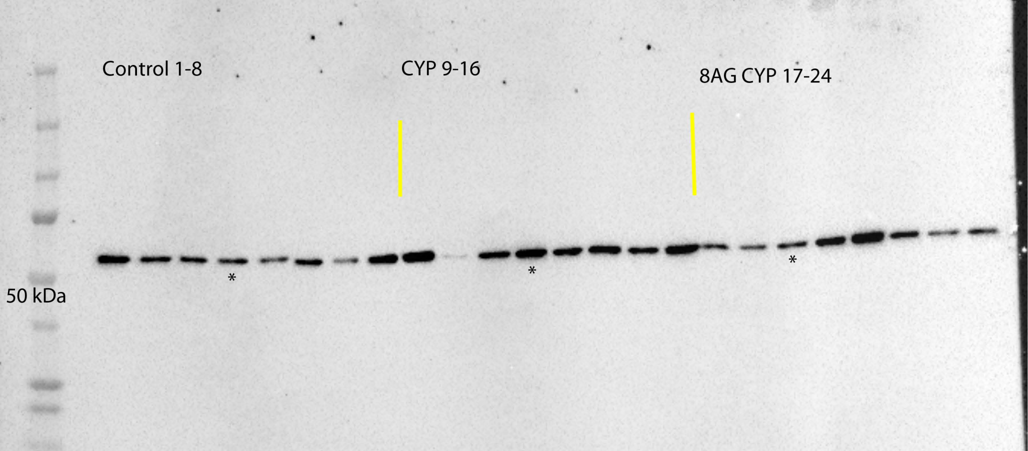

Full unedited blot (2 of 2) for figure 1M
MST3b (59kDa), Cell Signaling 4062, represented bands indicated by *

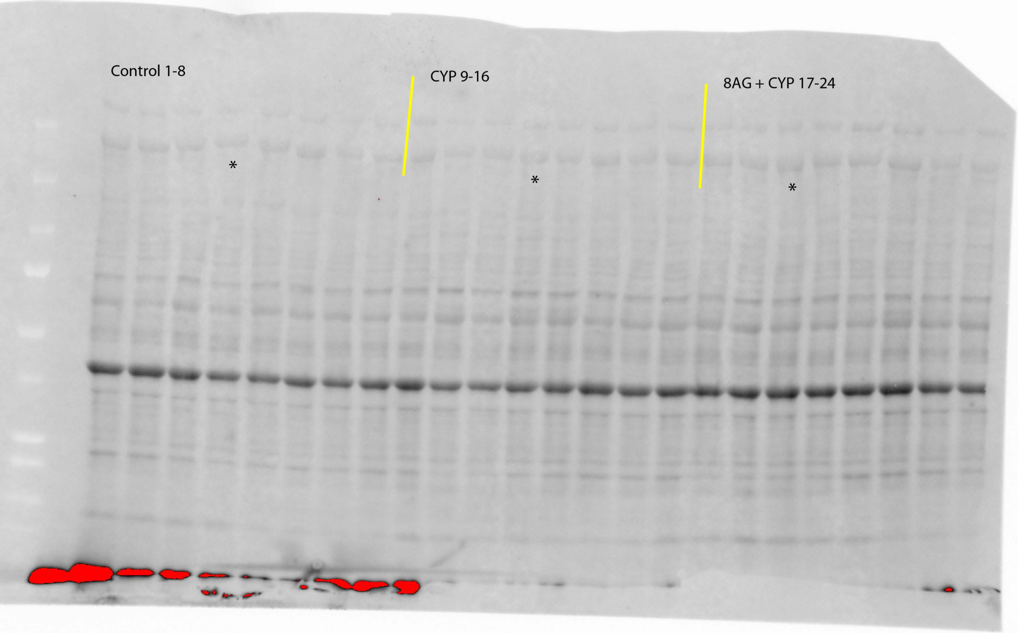

Full unedited stain free blot (2 of 2) for Figure 1M (MST3b)
Representative lanes marked by *

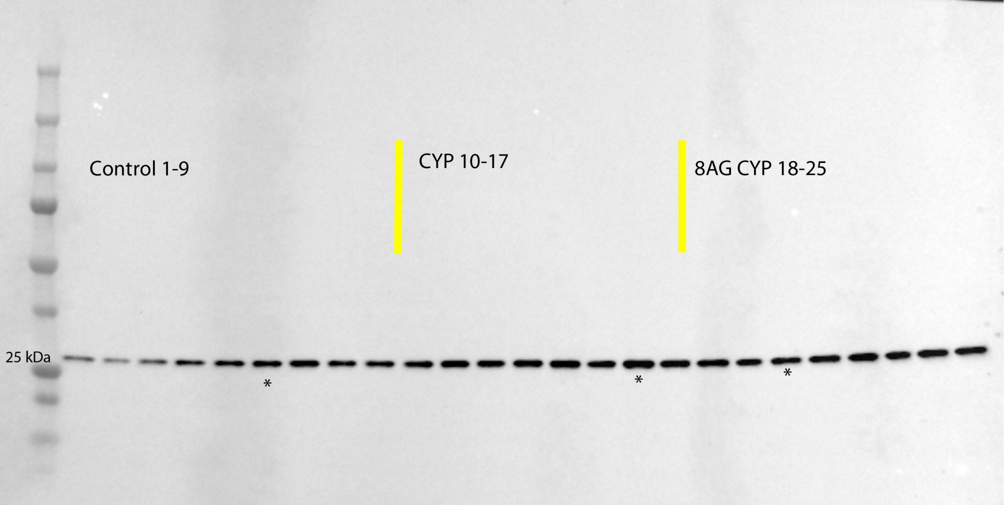

Full unedited blot (1 of 1) for Figure 1N
PNPase (32kDa), Atlas Antibodies HPA001625, representative bands marked by *

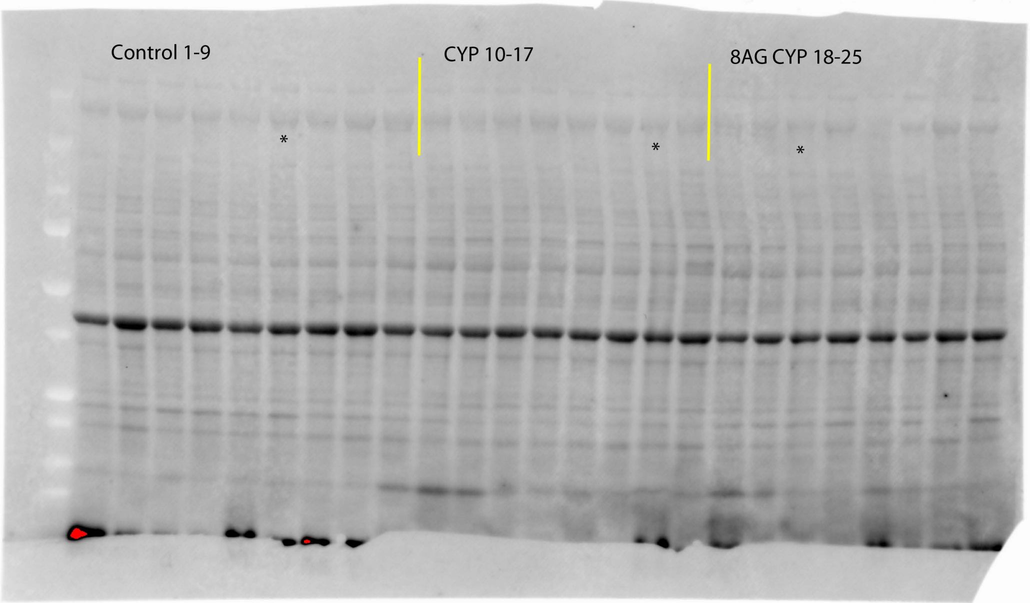

Full unedited stain free, total protein blot (1 of 1) for Figure 1N (PNPase)
Representative lanes marked by *

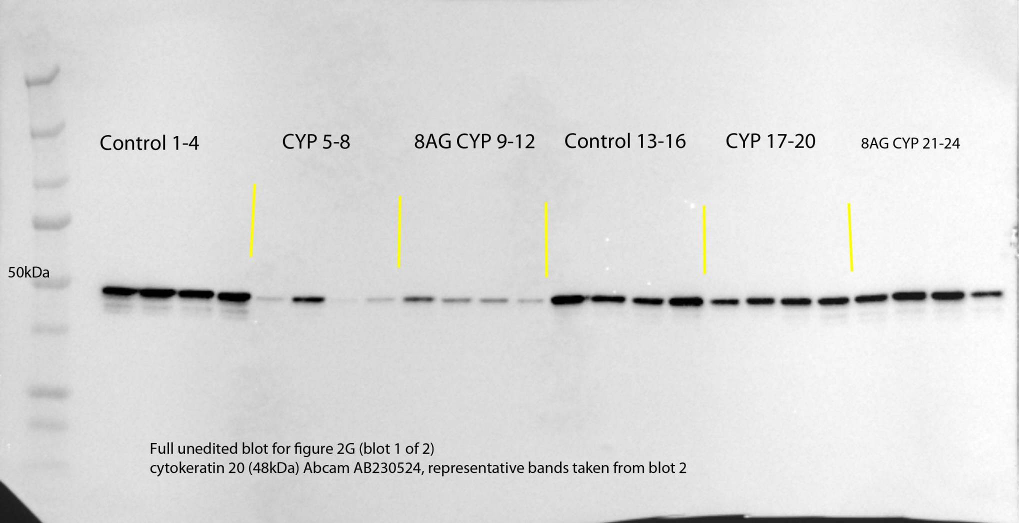

Full unedited blot for figure 2G (blot 1 of 2)
cytokeratin 20 (48kDa) Abcam AB230524, representative bands taken from blot 2

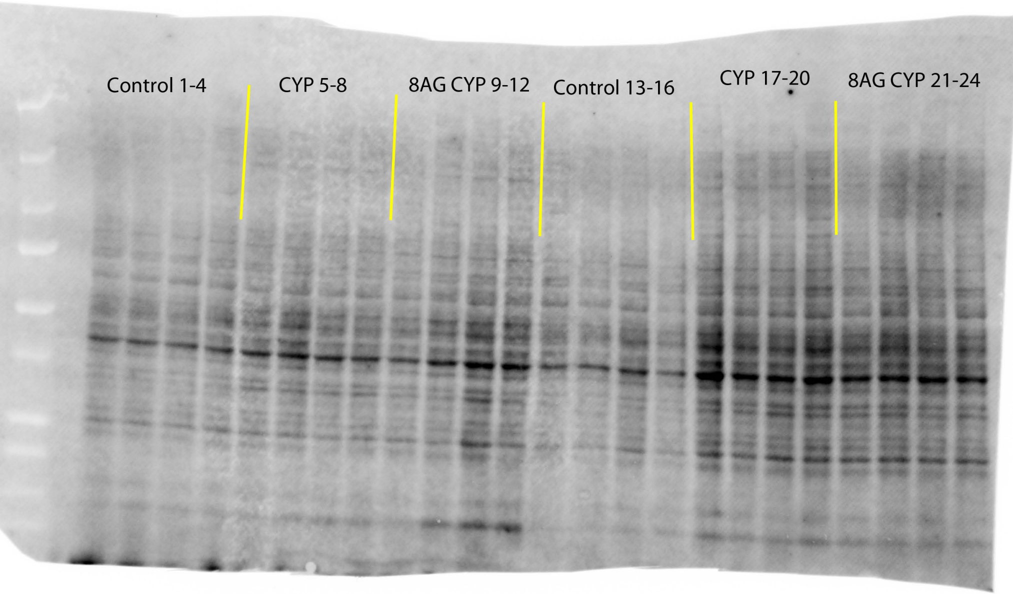

Full unedited stain free, total protein blot (blot 1 of 2) for Figure 2G (Cytokeratin 20)
Representative lanes taken from blot 2

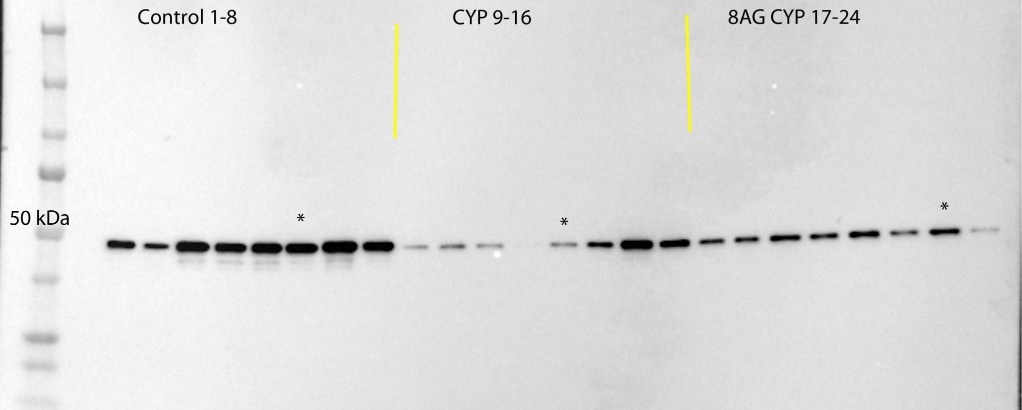

Full unedited blot (2 of 2) for figure 2G
cytokeratin 20 (48kDa) Abcam AB230524, representative bands marked with *

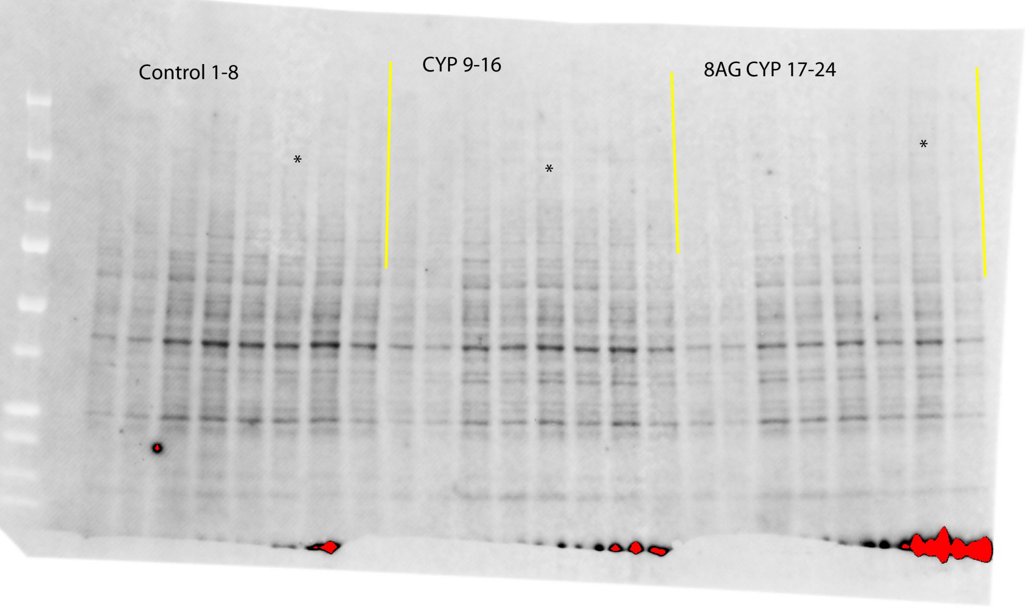

Full unedited stain free blot (2 of 2) for Figure 2G (Cytokeratin 20)
Representative lanes marked by *

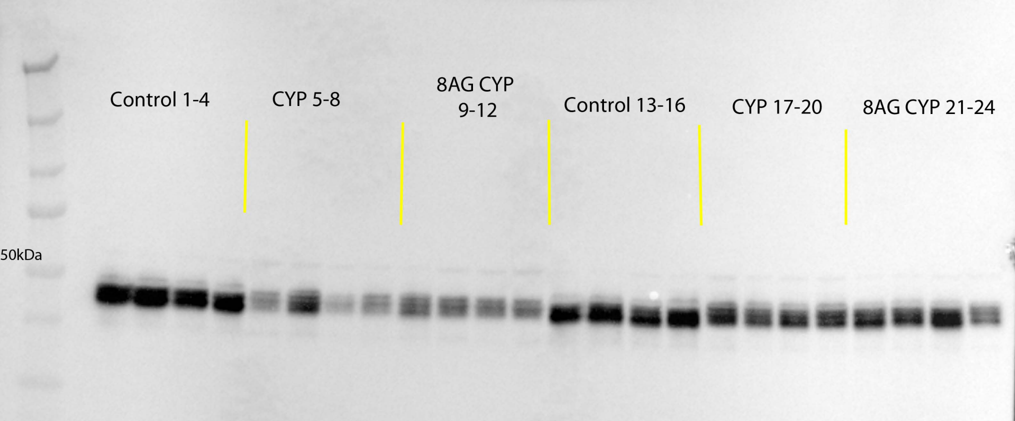

Full unedited blot (blot 1of 2) for figure 2H
predicted MW 31kDa, post-translationally glycosylated
uroplakin III Abcam AB231576, representative bands taken from blot 2

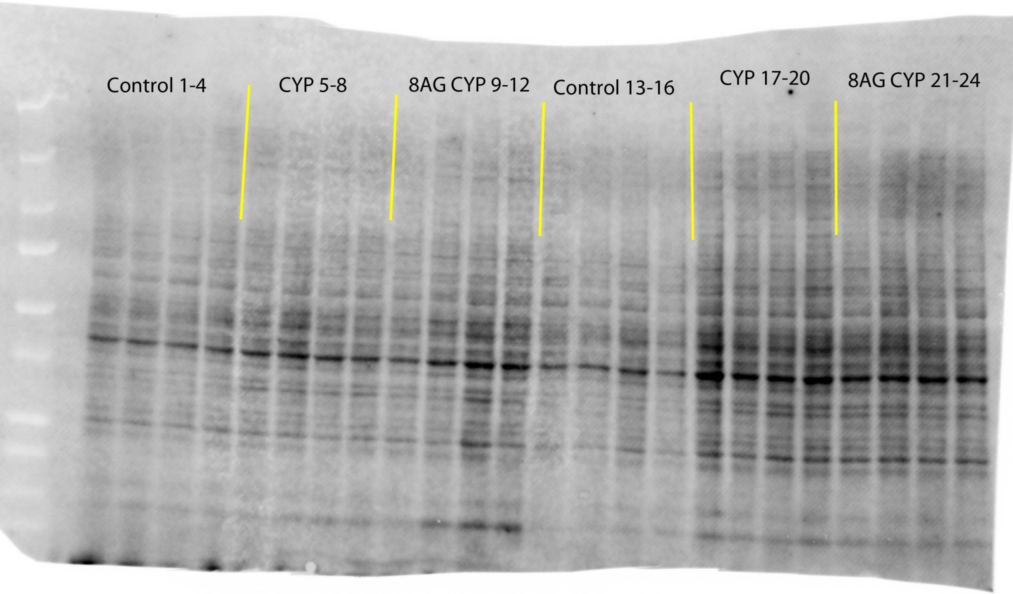

Full unedited stain free, total protein blot (blot 1 of 2) for Figure 2H (Uroplakin III)
Representative lanes taken from blot 2

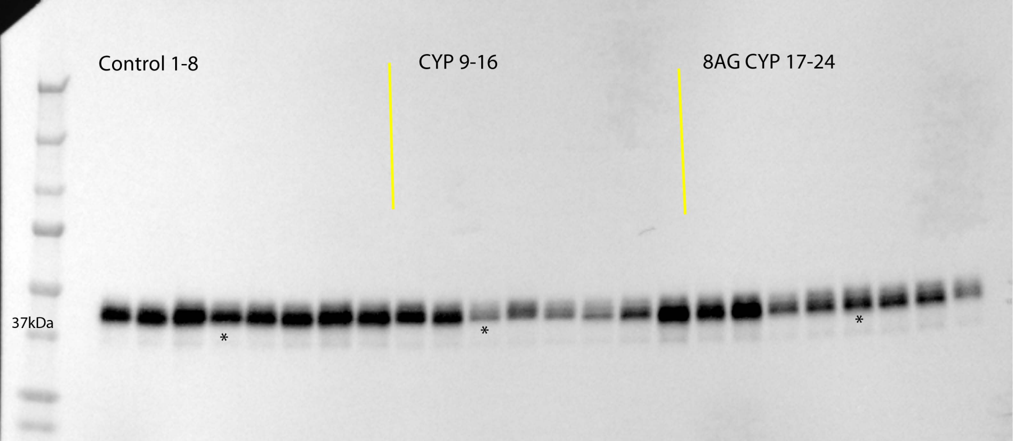

Full unedited blot (blot 2 of 2) for figure 2H
predicted MW 31kDa, post-translationally glycosylated
uroplakin III Abcam AB231576, representative bands indicated by *

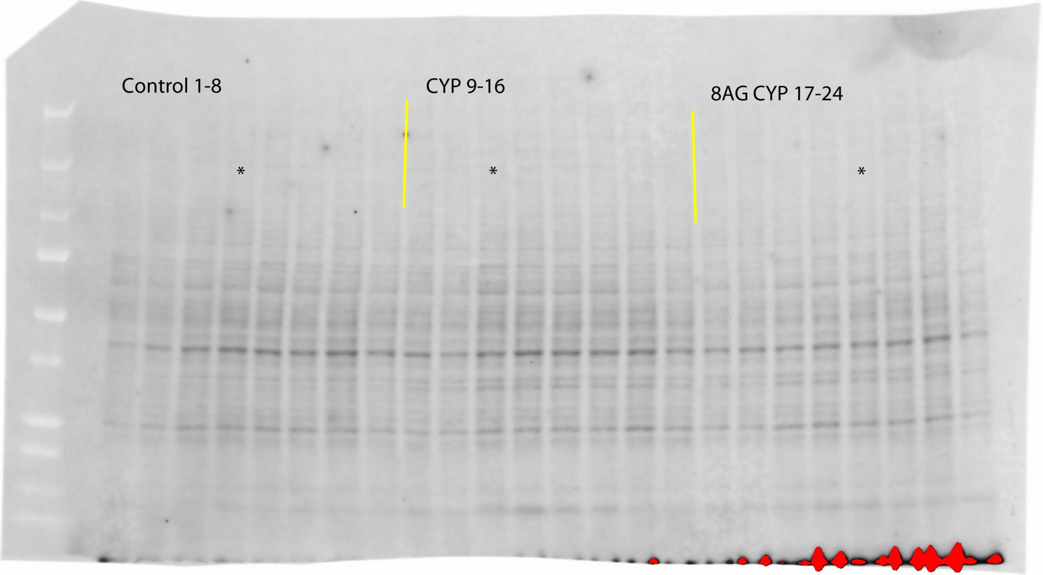

Full unedited stain free, total protein blot (blot 2 of 2) for Figure 2H (Uroplakin III)
Representative lanes marked by *

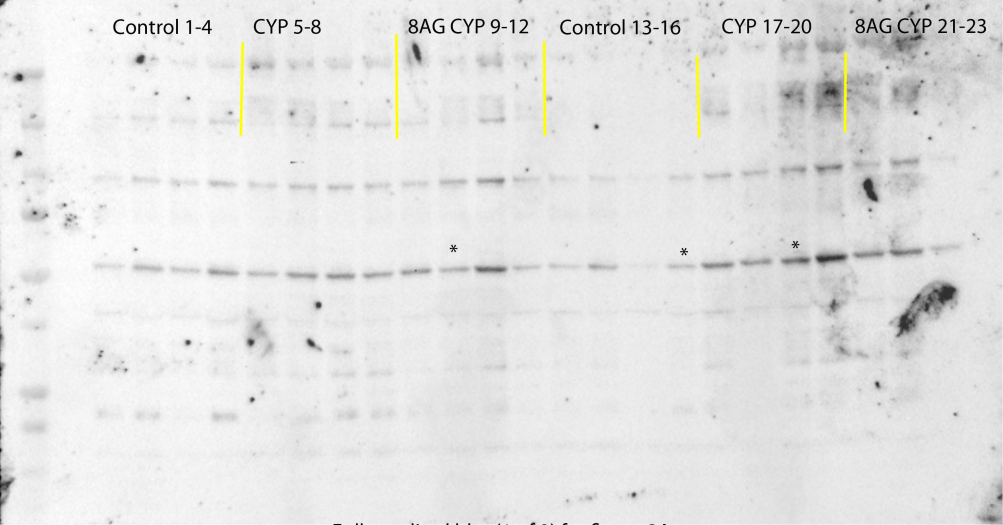

Full unedited blot (1 of 2) for figure 3A
Nitrotyrosine (multiple bands), Enzo life sciences BML-SA-468-100
representative bands indicated by *

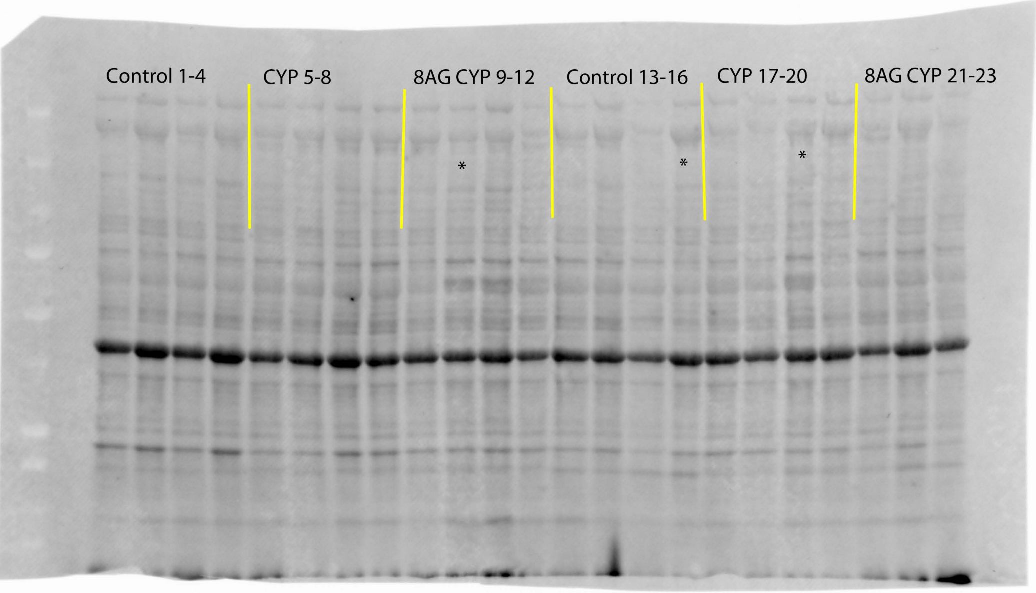

Full unedited stain free, total protein blot (blot 1 of 2) for Figure 3A (nitrotyrosine)
Representative lanes indicated by *

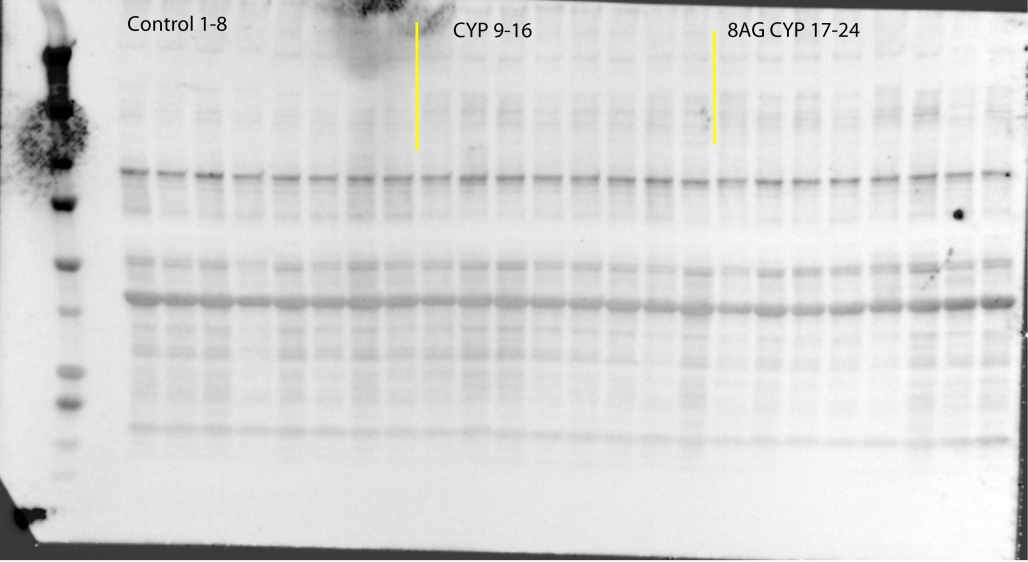

Full unedited blot (2 of 2) for figure 3A
Nitrotyrosine (multiple bands), Enzo Life Sciences BML-SA-468-100,
representative bands taken from blot 1

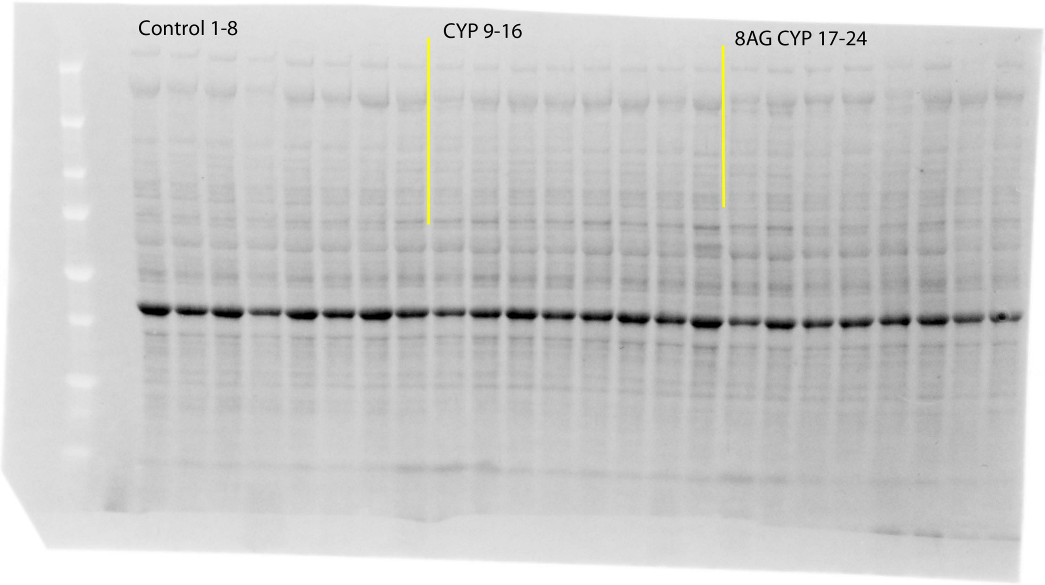

Full unedited stain free, total protein blot (blot 2 of 2) for Figure 3A (nitrotyrosine)
Representative lanes taken from blot 1

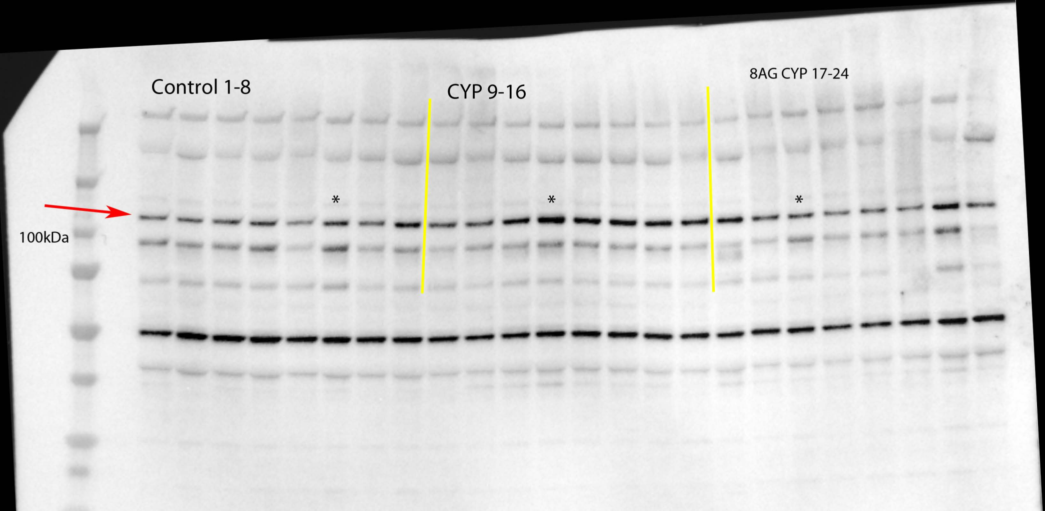

Full unedited blot (1 of 1) for figure 3B
NLRP3 (118kDa), Abcam AB214185, representative bands marked by *

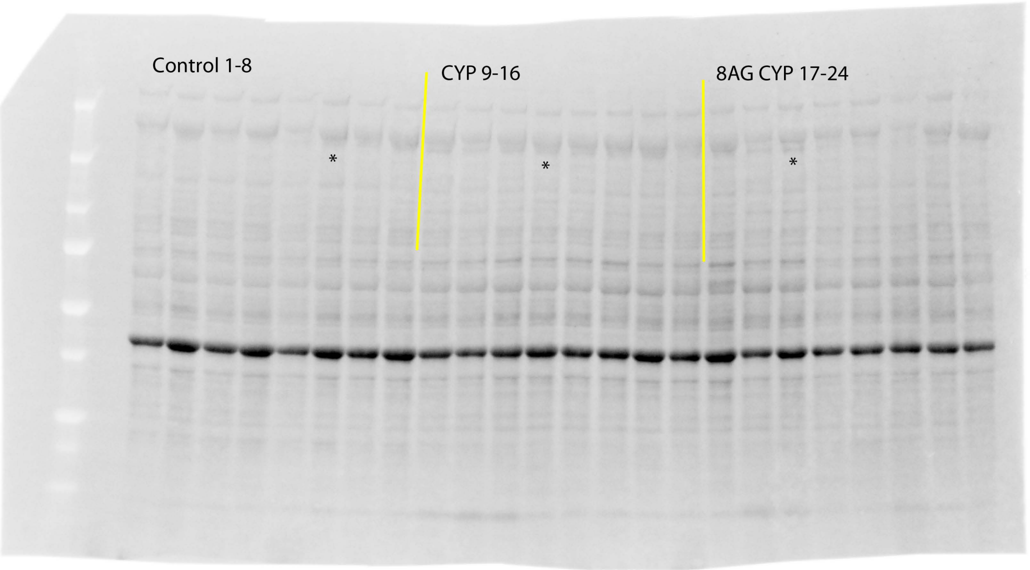

Full unedited stain free blot (1 of 1) for Figure 3B (NLRP3)
Representative lanes marked by *

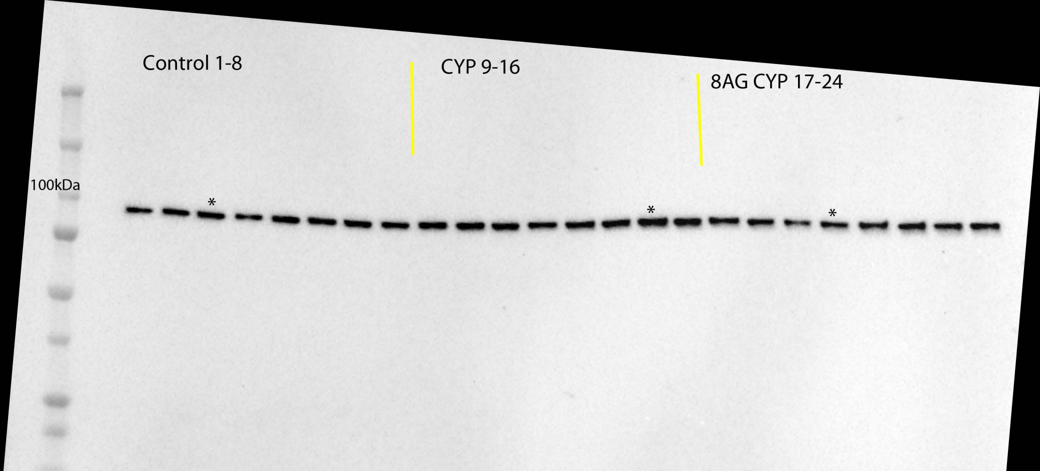

Full unedited blot (1 of 2) for figure 3C
TLR4 (95kDa) Santa Cruz SC-293072, representative bands marked by *

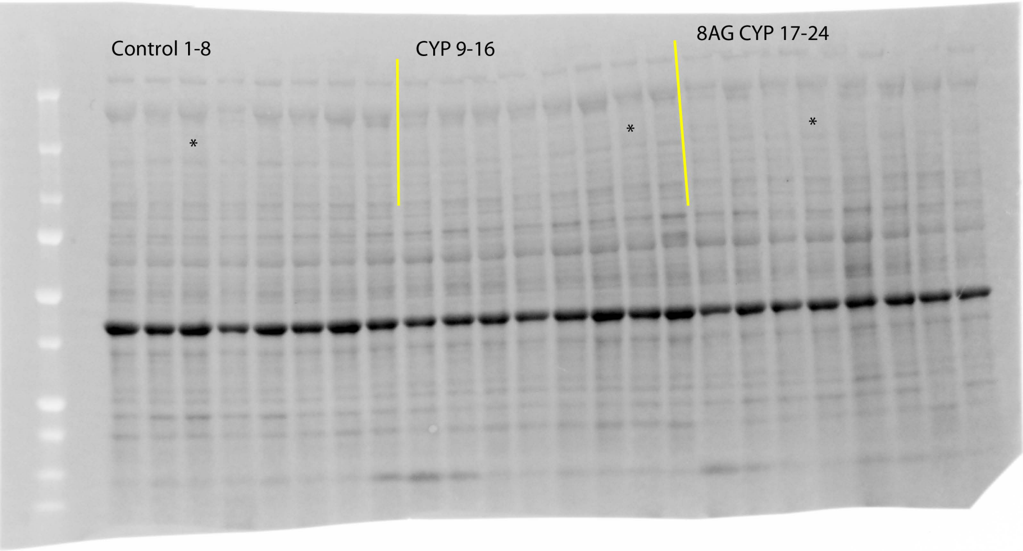

Full unedited stain free blot (1 of 2) for Figure 3C TLR4
Representative lanes indicated by *

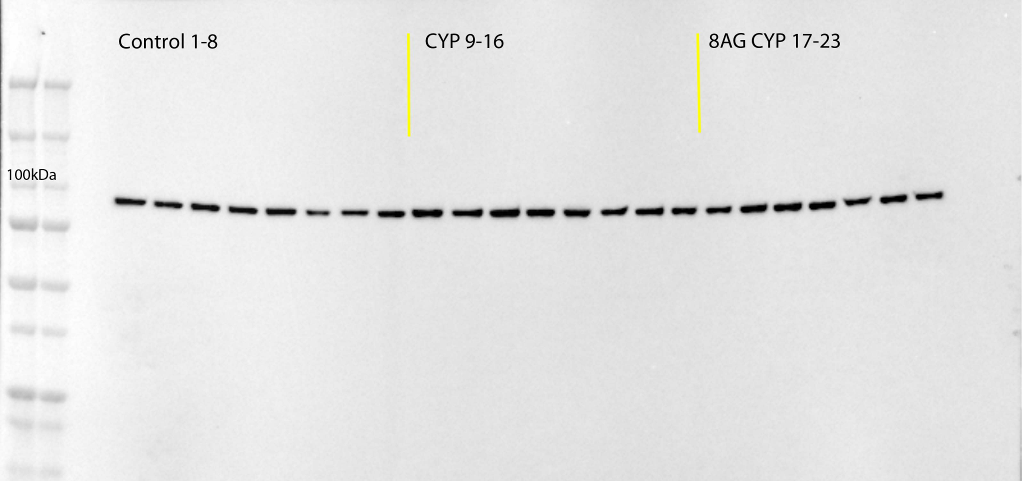

Full unedited blot (2 of 2) for figure 3C
TLR4 (95kDa) Santa Cruz SC-293072, representative bands taken from blot 1

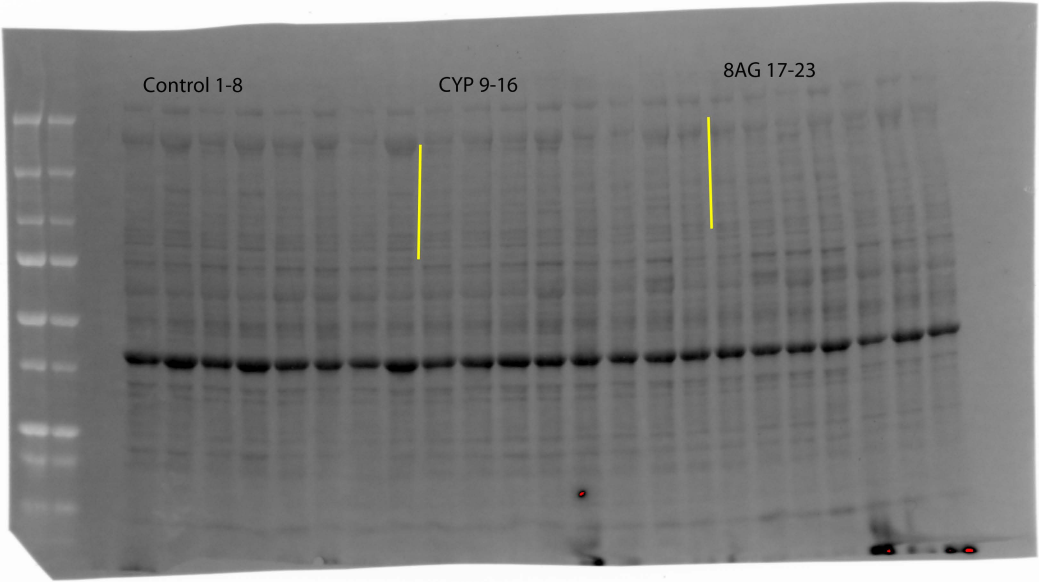

Full unedited stain free blot (2 of 2) for Figure 3C (TLR4)
Representative lanes taken from blot 1

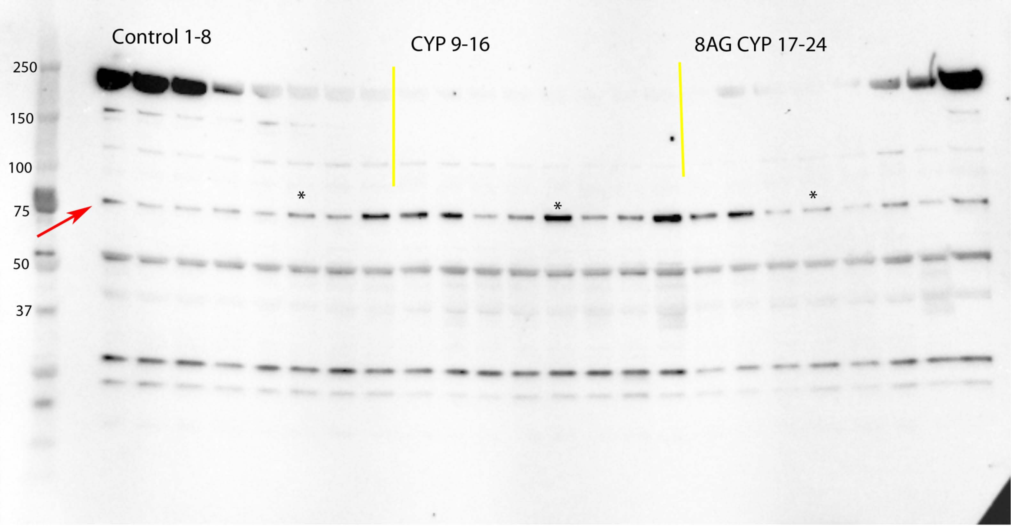

Full unedited blot (1 of 1) for figure 3D
HIF1α (80kDa) Abcam AB216842, representative bands marked by *

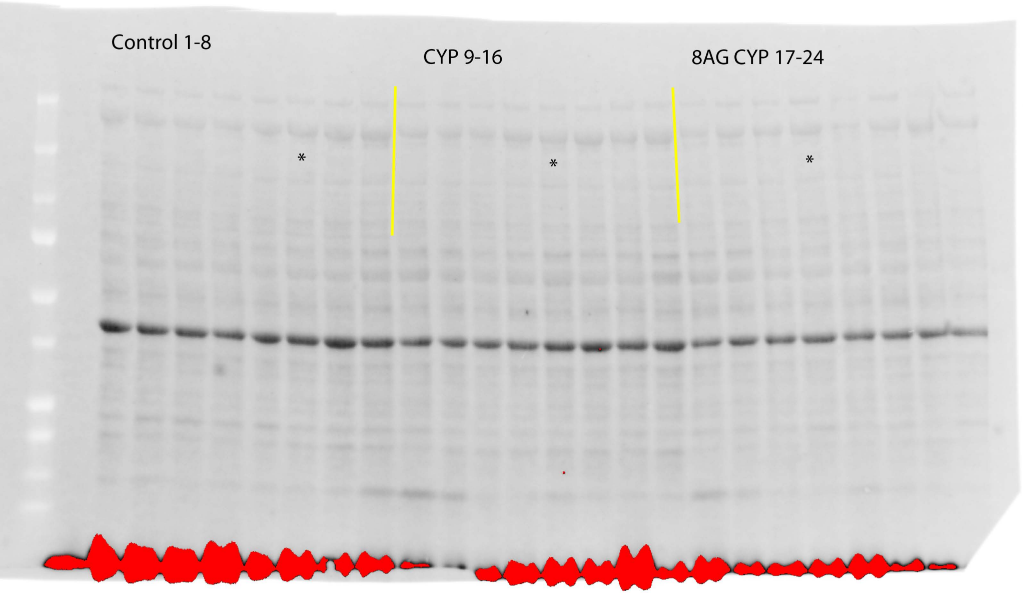

Full unedited stain free blot (1 of 1) for Figure 3D HIF-1alpha
Representative lanes indicated by *

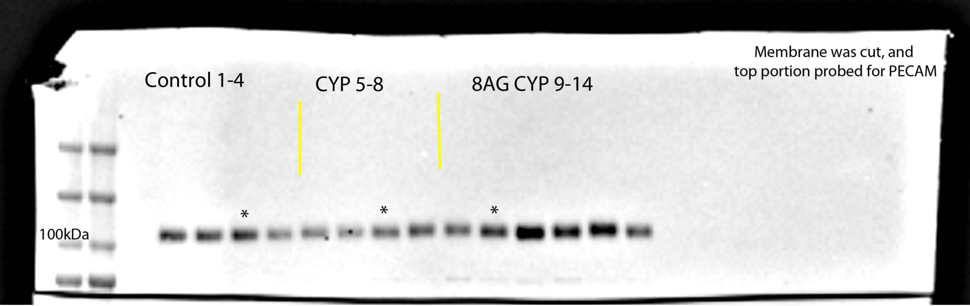

Full unedited blot (1 of 2) for figure 3E
PECAM (130kDa) Novus Biosciences NB100-2284, representative bands marked by *
membrane was cut and top portion probed for PECAM

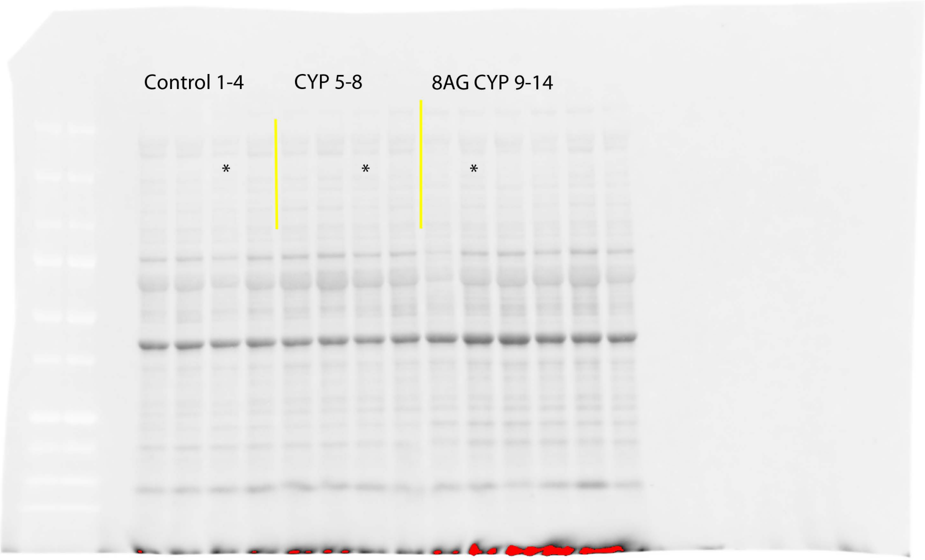

Full unedited stain free blot (1 of 2) for Figure 3E PECAM
Representative lanes indicated by *

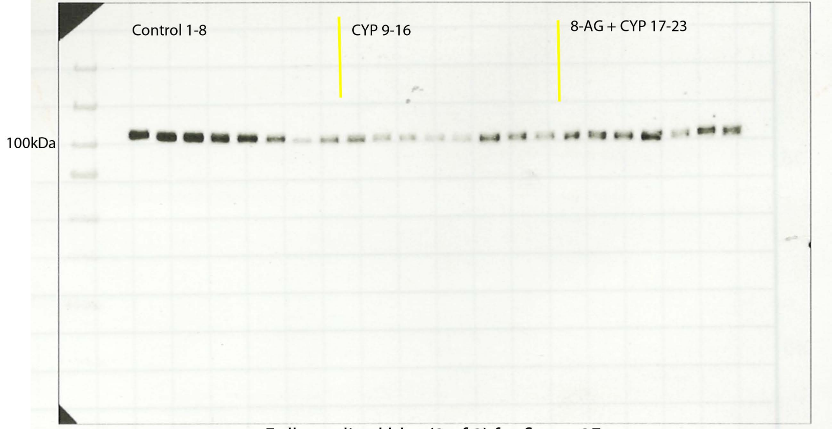

Full unedited blot (2 of 2) for figure 3E
PECAM (130kDa) Novus Biosciences NB100-2284,
representative bands taken from blot 1
